# Robust barcoding and identification of *Mycobacterium tuberculosis* lineages for epidemiological and clinical studies

**DOI:** 10.1186/s13073-020-00817-3

**Published:** 2020-12-14

**Authors:** Gary Napier, Susana Campino, Yared Merid, Markos Abebe, Yimtubezinash Woldeamanuel, Abraham Aseffa, Martin L. Hibberd, Jody Phelan, Taane G. Clark

**Affiliations:** 1 Faculty of Infectious and Tropical Diseases, London School of Hygiene & Tropical Medicine, Keppel Street, London, WC1E 7HT UK; 2 Armauer Hansen Research Institute, Addis Ababa, Ethiopia; 3 Department of Microbiology, Immunology and Parasitology, College of Health Sciences, Addis Ababa University, Addis Ababa, Ethiopia; 4 Hawassa University College of Medicine and Health Sciences, Hawassa, Ethiopia; 5 Faculty of Epidemiology and Population Health, London School of Hygiene & Tropical Medicine, London, WC1E 7HT UK

**Keywords:** Tuberculosis, Diagnostics, Profiling, SNPs, Barcoding, Mycobacteria tuberculosis complex

## Abstract

**Background:**

Tuberculosis, caused by bacteria in the *Mycobacterium tuberculosis* complex (MTBC), is a major global public health burden. Strain-specific genomic diversity in the known lineages of MTBC is an important factor in pathogenesis that may affect virulence, transmissibility, host response and emergence of drug resistance. Fast and accurate tracking of MTBC strains is therefore crucial for infection control, and our previous work developed a 62-single nucleotide polymorphism (SNP) barcode to inform on the phylogenetic identity of 7 human lineages and 64 sub-lineages.

**Methods:**

To update this barcode, we analysed whole genome sequencing data from 35,298 MTBC isolates (~ 1 million SNPs) covering 9 main lineages and 3 similar animal-related species (*M. tuberculosis* var. *bovis*, *M. tuberculosis* var. *caprae* and *M. tuberculosis* var. *orygis*). The data was partitioned into training (*N* = 17,903, 50.7%) and test (*N* = 17,395, 49.3%) sets and were analysed using an integrated phylogenetic tree and population differentiation (*F*_ST_) statistical approach.

**Results:**

By constructing a phylogenetic tree on the training MTBC isolates, we characterised 90 lineages or sub-lineages or species, of which 30 are new, and identified 421 robust barcoding mutations, of which a minimal set of 90 was selected that included 20 markers from the 62-SNP barcode. The barcoding SNPs (90 and 421) discriminated perfectly the 86 MTBC isolate (sub-)lineages in the test set and could accurately reconstruct the clades across the combined 35k samples.

**Conclusions:**

The validated 90 SNPs can be used for the rapid diagnosis and tracking of MTBC strains to assist public health surveillance and control. To facilitate this, the SNP markers have now been incorporated into the *TB-Profiler* informatics platform (https://github.com/jodyphelan/TBProfiler).

## Background

Tuberculosis, caused by bacteria in the *Mycobacterium tuberculosis* complex (MTBC), is a major global burden causing approximately ten million active cases and killing 1.5 million people in 2018 (www.who.int/tb). The MTBC consists of *Mycobacterium tuberculosis* sensu stricto (*Mtb*) (lineages 1, 2, 3, 4 and 7) and *M. tuberculosis* var. *africanum* (lineages 5 and 6; *M. africanum*), which cause human disease, but others including *M. tuberculosis* var. *bovis* affect predominantly animals [1]. Recently, new *Mtb* lineages (8, 9) have been proposed [2, 3]. The MTBC lineages vary in their geographic distribution and spread, being endemic in different locations around the globe, leading to the hypothesis that the strain types are specifically adapted to different human populations [4]. Lineage 2 is particularly mobile with evidence of recent spread from Asia to Europe and Africa. Lineage 4 is common in Europe and southern Africa, with regions of high TB incidence and high levels of HIV co-infection, whilst lineages 5, 6 and 7 appear isolated within West Africa and Ethiopia, respectively [1].

There is some evidence to suggest that MTBC lineages can determine the transmission, control, and clinical outcome of pulmonary and extra-pulmonary tuberculosis. In particular, variational phenotypes include differences in the emergence of drug resistance, transmissibility, virulence, host response, disease site and severity [5, 6]. Such phenotypes confer advantages for those MTBC lineages and may lead to an increased likelihood of disease spread and poorer prognosis for patients. Whether increased virulence is associated with poorer prognosis is unclear, with some studies reporting increased mortality risk with strains thought to be less virulent [7]. Of particular concern are the emergence of drug-resistant, multidrug-resistant (MDR-TB) and extensively drug-resistant (XDR-TB) strains, where Beijing strains show strong linear-resistance associations [8]. However, there is considerable inter-strain variation within lineages. For example, when comparing two different Beijing sub-lineages, the “ancient” (atypical) and “modern” (typical) strains show differences in geographical distribution, drug resistance and virulence patterns [9]. In particular, the “modern” sub-lineage is distributed worldwide and has been largely associated with MDR-TB and XDR-TB and hypervirulence [9].

Tracking the spread of lineages is of great importance in tuberculosis research and control. Rapid lineage identification enables the analysis of phenotypic associations, informs on likely provenance and can assist in the prediction of potential future outbreaks. The molecular barcoding of lineages and sub-lineages can be used to classify clinical isolates to aid in the evaluation of tools to control the disease, including therapeutics and vaccines, whose effectiveness may vary by strain type [1, 5]. Historically, strain identification has involved the genotyping of tandem repeats (e.g. spoligotypes) and large deletions (regions of difference (RDs)) [10], but these approaches are being replaced by methods analysing data from whole genome sequencing (WGS) technologies. These approaches include in silico spoligotyping and RD detection, the characterisation of lineage-associated single nucleotide polymorphisms (SNPs) and higher resolution methods such as core genome MLST [11]. SNP-based approaches can be applied in silico or implemented within a laboratory typing assay [12, 13]. Although the SNP-defined lineages do not offer the same resolution as using the whole genome, they provide a valuable insight into the epidemiology of circulating strains. A 62-SNP barcode was developed using WGS data for 1601 MTBC isolates and was the first to position samples within clades of a global phylogeny of 7 human lineages and 64 sub-lineages, covering all common strain types [1].

Here, we update the 62-SNP barcode using WGS for 35,298 MTBC isolates. In particular, we use WGS data for 17,903 (50.7%) isolates to reconstruct a global phylogeny, resulting in 30 new (sub-)lineages. This analysis led to the 62-SNP barcode being modified and extended to ninety robust SNPs to cover 90 MTBC (sub-)lineages or species, including animal-related *M. tuberculosis* var. *bovis* (*M. bovis*), *M. tuberculosis* var. *caprae* (*M. caprae*) and *M. tuberculosis* var. *orygis* (*M. orygis*), which are similar and sometimes misclassified. The new barcode was validated on the 17,395 (49.3%) remaining MTBC isolates. The ninety SNP markers have been incorporated into the *TB-Profiler* software (https://github.com/jodyphelan/TBProfiler) [14], which has been used to profile more than fifty thousand MTBC for strain types and drug resistance, and will thereby assist with barcode implementation for research and infection control activities.

## Methods

### Sample, raw data and sequence analysis

Illumina whole genome sequencing data was publicly available across 35,298 MTBC isolates, which encompassed *Mtb* lineages (1, 2, 3, 4 and 7), *M. africanum* (lineages 5 and 6), *M. bovis*, *M. caprae* and *M. orygis* [14], and the recently proposed lineages 8 [2] and 9 [3] (Additional file 1: Table S1). The data were convenience sampled with the first processed set (*n* = 17,903; 50.7%) serving as a training dataset, and the second set collated subsequently (*n* = 17,395; 49.3%) serving as a testing dataset (Additional file 1: Table S1). The test set covers all the sub-lineages in the training set with at least 10 isolates (range 10–917), except (sub-)lineages 3.1.2.2, 4.6.2.1, 8 and 9, but for these the number of training samples is relatively small.

All raw sequences were trimmed using *trimmomatic* software [15] (v0.36, parameters: PE -phred33 LEADING:3 TRAILING:3 SLIDINGWINDOW:4:20 MINLEN:36). Trimmed reads were then aligned with *BWA-MEM* software [16] (v0.7.17-r1188, default parameters) using the H37Rv reference sequence (Genbank accession number: NC_000962.3). Alignments from *BWA-MEM* were converted to “bam” format and sorted using *samtools* software [17] (v1.9, default parameters). SNPs were identified by applying *BCFtools* [17] (v1.9, mpileup parameters: default, call parameters: -mv) and *GATK* software [18] (version: 4.1.3.0) using the HaplotypeCaller function (parameters: -ERC GVCF). Individual sample “vcf” files were merged using *GATK* GenomicsDBImport (default parameters) and *GATK* CombineGVCFs (default parameters) to perform joint calling using all samples. The resulting multi-sample vcf file was filtered to remove indels and heterozygous calls and monomorphic SNPs. A multi-FASTA file containing all isolates was generated from the filtered SNP file (*N* = 1,014,762 SNPs; training 620,652 SNPs; test 533,152 SNPs) and H37Rv reference genome using *bedtools* (v2.28.0) [19] and in-house python scripts. The regions of difference (RDs) were detected using *delly* software [20] and confirmed using de novo assembly by applying *Spades* software [21]. Spoligotypes were called using *spolpred* software [22].

### Principal component analysis and phylogenetic tree

Distance matrices and the principal components of the multi-FASTA files were computed with *Plink* software (v1.90b4; https://www.cog-genomics.org/plink2) [23]. The distance matrices were used for the new cluster identification. Maximum likelihood phylogenetic trees were constructed from the multi-FASTA file using *IQ-TREE* (v1.6.12) (http://www.iqtree.org/) [24]. A general time reversible model with rate heterogeneity set to a discrete Gamma model and an ascertainment bias correction were used (parameters *-m GTR+G+ASC*), with 1000 bootstrap samples used to measure branch quality and robustness. Phylogenetic trees were generated for all MTBC isolates, as well as for each main lineage separately. The resulting Newick-formatted tree files were visualised and annotated with metadata in *iTOL* (v5.2; https://itol.embl.de/) [25]. These metadata included the 62-SNP barcode sub-lineage predictions [1], allowing for the rapid identification of outliers. By annotating the branches with ancestral mutations, it was possible to inform on SNP markers for barcoding.

### Lineage revision and new sub-lineage identification

The visual inspection of the phylogenetic trees (and principal component analysis plots) revealed that some pre-existing (sub-)lineages (as defined using the 62-SNP barcode) could be merged or split, as well as new ones created. The original 62-SNP barcode was constructed to reflect the original strain-type families used by researchers based on spoligotypes and RDs. We sought to analyse the phylogenetic tree to further divide these clades where obvious splits in the phylogeny existed. To aid in old lineage revision and new lineage identification, phylogenetic trees relating to lineages 1 to 9 and animal strains were analysed using a semi-automated procedure. Each tree was traversed (and each clade inspected) from root to tip using the *ETE3 Toolkit* (v3.1.1) package in Python3 (http://etetoolkit.org/) [26]. We identified metrics and parameters such as branch bootstrap support values and intra/inter-cluster SNP distances to determine splits in the tree, which led to clusters that are separated by long branch lengths from other isolates. Whilst traversing, the following criteria had to be met to establish clades leading to new or revised sub-lineages: (1) a minimum clade size of 20, with a branch supported by a bootstrap value of > 95; (2) differences in the distributions of SNP distances where comparing the isolates within and outside the clade, using a Welch *t* test assuming unequal variances [27] (*P* < 0.05) and a Cohen’s *d* effect size [28] (*d* > 0.5); (3) the ratio of the branch length of the clade compared to the mean branch length of its descendants (ratio > 1); (4) estimation of the number of clade-informative SNPs, requiring at least 10 SNPs with a fixation index (*F*_ST_) [29] value of 1; (5) confirmation of the clade through visual inspection of the tree. Each of the parameter thresholds was based on established cut-offs or determined using standard point of inflection methods [1]. The population differentiation *F*_ST_ statistic assigns a strength of association between each SNP and (sub-)lineage, with a score of 1 indicating that the SNP allele is fixed in the sub-lineage of interest and not present outside that group. Using the five criteria led to the addition of 87 (27 new) sub-lineages or lineages (including 8 and 9), or changing the branch position of established others (e.g. 1.2 and 1.1.1.1) (see Additional file 1: Fig. S1). The *SNP-IT* tool for identifying species in MTBC [30] was applied to the *M. bovis*, *M. orygis* and *M. caprae* isolates (*N* = 110; test set), and three barcoding SNPs were required for these mycobacteria. The overall number of (sub-)lineages or species covered was 90.

### Barcoding SNPs

To ensure that the required 90 clade-specific mutations (“potential barcoding SNPs”, all with *F*_ST_ = 1) were robust, where possible, we retained synonymous SNPs in essential genes [31], and excluded those in drug resistance loci (from *TB-Profiler* [14]) and non-essential PE/PPE gene families [32]. From those retained “robust” SNPs (*n* = 421), a minimal set of one per lineage included preferentially those already present in the 62-SNP barcode [1] and, if not possible, (arbitrarily) the lowest position was chosen. The gene functional categories were extracted from *Tuberculist* (tuberculist.epfl.ch), and the frequency of ontologies across all potential barcoding, robust and minimal SNPs, was assessed for differences across lineage using the chi-squared tests.

### Validation of lineage barcode

To validate the final set of robust 421 clade-defining SNPs (Additional file 1: Table S2), the 17,395 samples in the testing set (with 572,021 SNPs) were used. The (sub-)lineage of these samples was predicted with *TB-Profiler* [14]. At the same time, a phylogenetic tree was reconstructed of the training and test samples together using *FastTree2* software [33]. To assess the sensitivity and specificity of the predictions, this tree was traversed in the *ETE3 Toolkit*, and test samples were examined for their presence in the clades defined by the training dataset.

## Results

### MTBC isolates, SNPs and phylogeny

**Fig. 1:**
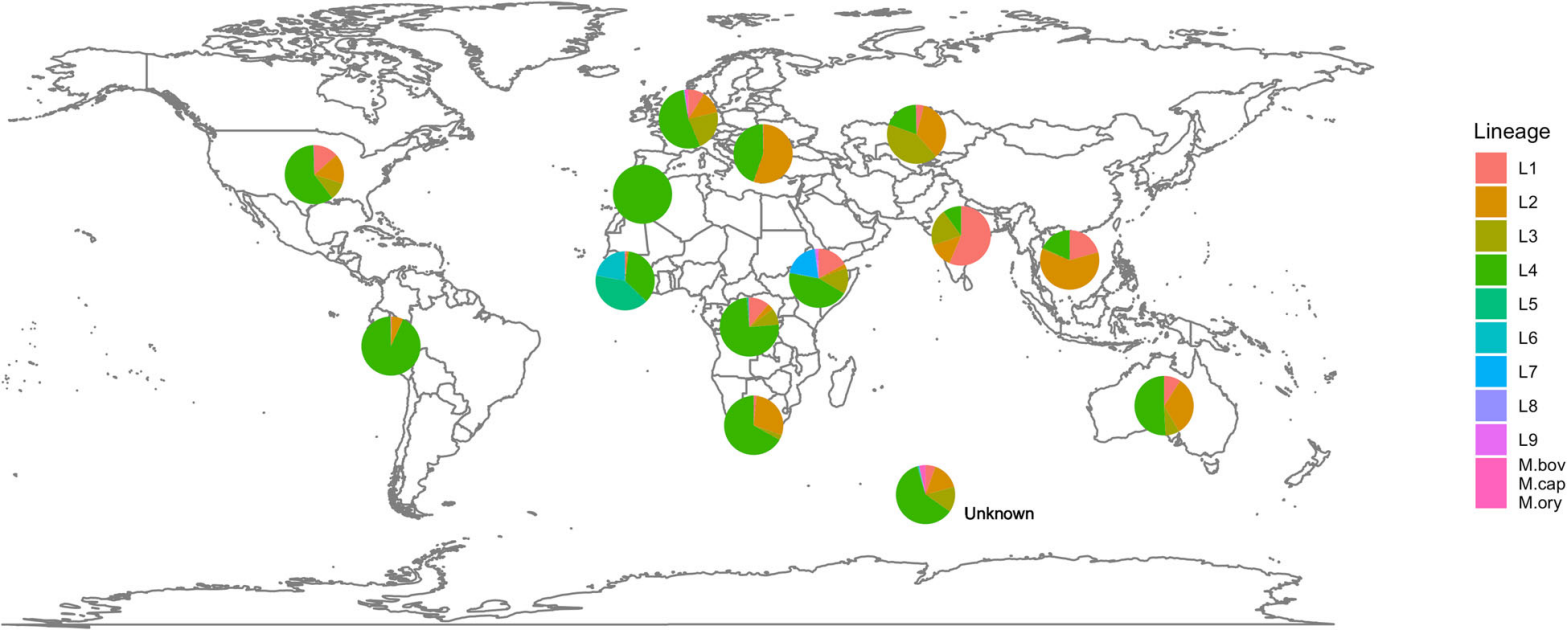
The global distribution of the 35,298 *Mycobacterium tuberculosis* complex study isolates

Across a total of 35,298 MTBC isolates with sequencing data, we identified 1,014,762 high-quality SNPs. The isolates represented all MTBC lineages (1–9), *M. bovis*, *M. orygis* and *M. caprae*, but the majority were from lineages 4 (51.6%), 2 (25.2%), 3 (11.1%) and 1 (9.5%), with the frequency of others being at most 1% (Additional file 1: Table S1). Whilst it is a convenience set of sampled isolates, the geographical distribution of the lineages was as expected, with lineage 2 dominating in Southeast Asia, lineages 1 and 3 predominant in South Asia, lineage 4 abundant in Europe, Americas and Africa and lineages 5 and 6 present in West Africa (Fig. 1). The East Asian lineage 2 had the highest frequency of MDR-TB isolates (36.2%), driven by a higher prevalence in the Beijing sub-lineage (lineage 2.2; 36.5%) compared to the Manu ancestor or proto-Beijing strain type (lineage 2.1, 19.8%) (Table 1).

**Table 1:** *Mycobacterium tuberculosis* complex lineages and sub-lineages across the 35,298 isolates

Lineage	No. training (test)	No. countries train (test)	% MDR-TB	No. transmission [clusters]	Potential barcoding SNPs*	Robust SNPs**
1	2162 (1203)	25 (42)	7.8	354 [130]	344	17
1.1	1487 (530)	19 (36)	5.5	218 [82]	23	2
1.1.1	706 (170)	8 (16)	3.8	60 [25]	41	5
1.1.1.1	358 (120)	5 (9)	2.1	28 [11]	52	3
1.1.2	459 (278)	15 (25)	9.0	83 [31]	109	3
1.1.3	299 (80)	11 (16)	3.2	73 [25]	42	2
**1.1.3.1**	84 (31)	7 (13)	3.5	10 [4]	68	2
**1.1.3.2**	155 (33)	7 (7)	1.1	57 [18]	113	6
**1.1.3.3**	32 (7)	5 (4)	10.3	4 [2]	36	2
**1.2**	309 (550)	13 (21)	7.5	40 [16]	60	2
1.2.1	28 (44)	3 (7)	6.9	6 [2]	78	5
1.2.2	277 (505)	13 (18)	7.5	34 [14]	159	8
**1.2.2.1**	244 (453)	12 (18)	6.9	34 [14]	34	1
**1.3**	366 (122)	16 (19)	18.0	96 [32]	71	2
**1.3.1**	88 (25)	7 (11)	10.6	20 [7]	50	4
**1.3.2**	278 (97)	16 (17)	20.3	76 [25]	83	4
2	4556 (4322)	45 (56)	36.2	1778 [413]	72	4
2.1	95 (41)	6 (9)	19.8	27 [10]	172	4
2.2	4461 (4281)	45 (56)	36.5	1751 [403]	79	17
2.2.1	4239 (4007)	45 (56)	35.1	1632 [389]	17	2
2.2.1.1	338 (443)	19 (18)	28.0	98 [40]	6	2
2.2.1.2	29 (21)	6 (9)	36.0	10 [3]	5	1
2.2.2	222 (273)	16 (15)	59.0	119 [14]	54	4
3	2654 (1271)	24 (31)	13.4	847 [242]	166	8
**3.1**	715 (362)	15 (22)	9.5	372 [80]	1	1
3.1.1	387 (280)	11 (16)	6.2	243 [43]	17	2
3.1.2	295 (69)	13 (8)	14.3	124 [35]	8	2
3.1.2.1	98 (25)	8 (7)	19.5	25 [12]	15	7
3.1.2.2	48 (0)	3 (0)	0	36 [2]	85	6
**3.2**	89 (31)	6 (9)	10.0	31 [7]	85	2
4	8320 (9883)	44 (99)	18.5	3109 [731]	94	3
4.1	2594 (2325)	35 (64)	18.5	1043 [191]	58	3
4.1.1	889 (482)	20 (27)	18.1	403 [72]	30	13
4.1.1.1	210 (158)	14 (16)	9.5	92 [20]	39	2
4.1.1.2	55 (44)	4 (6)	2.0	33 [3]	92	2
4.1.1.3	579 (247)	18 (23)	22.4	266 [44]	58	3
**4.1.1.3.1**	207 (13)	3 (3)	9.6	158 [5]	46	3
4.1.2	1612 (1743)	32 (61)	17.3	622 [113]	13	1
4.1.2.1	1383 (1087)	32 (60)	22.5	563 [96]	49	3
**4.1.2.1.1**	231 (18)	1 (1)	97.6	221 [2]	73	3
**4.1.3**	28 (70)	7 (10)	57.1	4 [2]	124	3
**4.1.4**	24 (12)	8 (7)	38.9	10 [2]	60	4
4.2	481 (532)	23 (26)	28.0	87 [32]	116	8
4.2.1	206 (240)	13 (20)	28.3	34 [13]	26	2
**4.2.1.1**	54 (148)	9 (10)	6.9	2 [1]	36	2
4.2.2	274 (288)	20 (18)	28.1	53 [19]	20	2
4.2.2.1	74 (41)	10 (6)	45.2	22 [7]	26	2
**4.2.2.2**	120 (139)	11 (14)	27.8	15 [7]	31	10
4.3	2507 (2928)	30 (75)	23.1	993 [244]	38	2
4.3.1	58 (67)	7 (15)	6.4	40 [3]	28	1
**4.3.1.1**	37 (2)	3 (1)	0.0	36 [1]	52	2
4.3.2	409 (1200)	16 (21)	7.2	75 [32]	75	1
4.3.2.1	291 (917)	6 (7)	3.7	50 [23]	55	4
4.3.3	648 (810)	25 (57)	41.3	210 [66]	33	1
4.3.4	1366 (807)	23 (45)	24.1	664 [142]	8	1
4.3.4.1	194 (170)	14 (30)	28.9	49 [14]	19	4
4.3.4.2	1170 (635)	22 (34)	23.1	614 [128]	26	1
4.3.4.2.1	877 (287)	13 (18)	5.6	457 [103]	11	1
4.4	560 (1059)	24 (29)	15.7	190 [63]	37	2
4.4.1	420 (861)	22 (25)	16.0	149 [48]	38	4
4.4.1.1	379 (755)	21 (24)	17.8	136 [44]	16	1
**4.4.1.1.1**	75 (206)	5 (4)	19.6	22 [9]	60	3
4.4.1.2	39 (106)	8 (6)	1.4	13 [4]	95	9
4.4.2	112 (181)	7 (9)	14.7	33 [13]	7	2
4.5	293 (357)	17 (17)	15.7	49 [22]	50	1
4.6	340 (442)	21 (25)	22.1	139 [39]	12	1
4.6.1	73 (296)	9 (12)	29.8	24 [8]	53	3
4.6.1.1	29 (126)	6 (7)	1.3	14 [3]	22	1
4.6.1.2	40 (154)	9 (11)	54.6	10 [5]	37	1
4.6.2	164 (89)	16 (17)	15.4	65 [20]	22	1
4.6.2.1	2 (0)	1 (0)	0	2 [1]	45	2
4.6.2.2	150 (89)	14 (17)	15.9	60 [18]	106	6
**4.6.3**	23 (9)	3 (4)	0	20 [3]	135	3
**4.6.4**	23 (7)	5 (4)	50.0	10 [2]	49	1
**4.6.5**	23 (18)	5 (5)	19.5	9 [3]	8	2
4.7	158 (200)	18 (23)	10.3	56 [20]	10	3
4.8	1051 (1807)	29 (55)	7.8	419 [88]	17	1
**4.8.1**	63 (90)	7 (4)	22.2	21 [5]	46	3
**4.8.2**	116 (5)	3 (2)	0	113 [1]	42	2
**4.8.3**	21 (3)	1 (1)	0	19 [1]	34	1
4.9	243 (141)	14 (22)	12.5	114 [24]	37	3
**4.9.1**	74 (15)	6 (3)	5.6	44 [1]	49	3
5	26 (255)	6 (12)	14.6	2 [1]	460	13
6	32 (135)	6 (13)	3.6	5 [2]	214	10
7	38 (26)	3 (2)	0	3 [1]	837	38
**8**	2 (0)	1 (0)	0	0 [0]	888	43
**9**	3 (0)	1 (0)	0	0 [0]	160	5
***M. bovis***	81 (281)	9 (12)	0.8	42 [11]	93	3
***M. caprae***	3 (7)	2 (3)	0	0 [0]	225	5
***M. orygis***	26 (12)	4 (4)	0	0 [0]	743	28
Totals	17,903 (17,395)	165 (269)	21.0	6140 [1531]	8128	421

Bolded are changes from the barcode in reference [1]—either new sub-lineages or new barcoding SNPs; MDR-TB multidrug-resistant TB, which is resistant to at least rifampicin and isoniazid drugs. *All potential barcoding SNPs (*F*_ST_ = 1). **Final robust SNP set, based on synonymous changes in essential and non-drug resistance genes only (except 12 sub-lineages which had no informative SNPs in essential genes; see Additional file 1: Table S2)

**Fig. 2:**
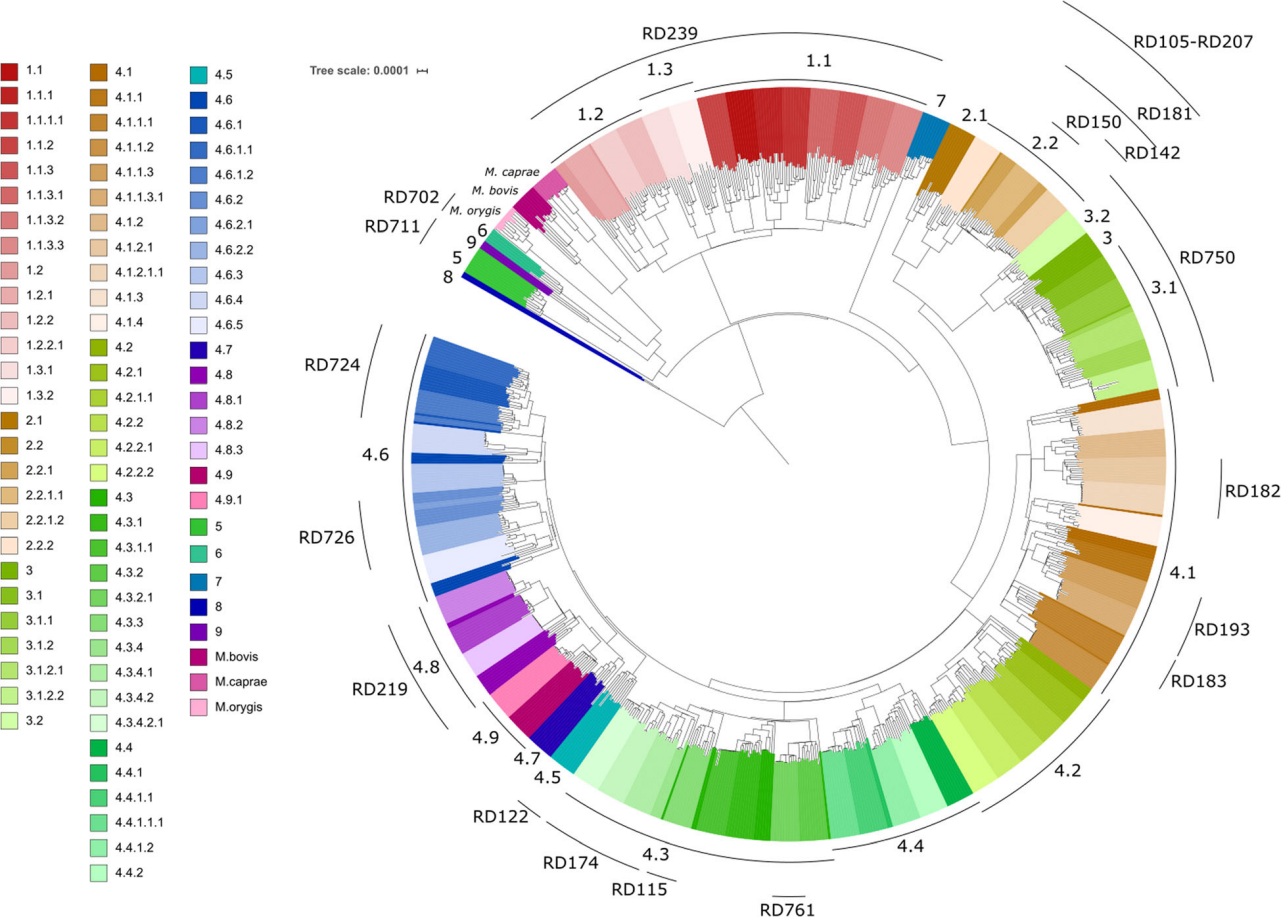
Phylogenetic tree of *Mycobacterium tuberculosis* complex isolates. A representative tree with a maximum of 10 isolates per sub-lineage (important regions of difference (RDs) are also highlighted)

The 35k isolates were split into training (*N* = 17,903, 50.7%; all MTBC; 620,652 SNPs) and test (*N* = 17,395, 49.3%, all MTBC except lineages 8 and 9; 572,021 SNPs) datasets (Table 1; Additional file 1: Table S1). A phylogenetic tree was constructed on the training isolates and confirmed the clustering by lineage and sub-lineages (Fig. 2). Similarly, a principal component analysis of the 35k isolates using the ~ 1 million SNPs revealed the expected clustering by lineage or species (Additional file 1: Fig. S1(a)). Phylogenetic trees were constructed for each lineage separately and confirmed the sub-lineage and strain-type clustering (Additional file 1: Fig. S1(b)-(f)). However, by assessing the fine-scale clustering of sub-lineages predicted by the 62-SNP barcode, outlying samples were revealed and suggested a need for the re-positioning of mutations underlying the clades or, alternatively, the creation of new sub-lineages that were on long branches (Additional file 1: Fig. S12(b, c)). In some cases, new sub-lineages reflected existing RD- or spoligotype-based strain classifications which were imperfectly or not captured using the 62-SNP barcode (see Additional file 1: Fig. S2 (d,e)).

### Barcoding SNPs

By traversing the whole MTBC and lineage-based phylogenetic trees using a semi-automated algorithm, it was possible to modify sub-lineages within the flexible nomenclature structure of the previous barcode [1], as well as define clade-informative SNPs. The phylogenetic analyses characterised 27 additional (sub-)lineages covering lineages 1 (8), 3 (2), 4 (15), 8 (1) and 9 (1). The final number of (sub-)lineages in *Mtb* was 85 (L (ineage)1 16, L2 7, L3 7, L4 52, L7 1, L8 1, L9 1) and *M. africanum* was 2 (L5 1, L6 1) (Table 1; Fig. 2), requiring 87 SNP markers. A further three SNP markers were required to discriminate *M. bovis*, *M. caprae* and *M. orygis*, which have highly similar mycobacterial genomes, and therefore, their accurate typing will greatly assist with the misclassification of *M. bovis* infections.

To find informative SNPs for each of the 90 MTBC clades, we used the population differentiation metric *F*_ST_ to identify mutations that were only present in the isolates in the selected (sub-)lineage of interest (*F*_ST_ = 1). We identified 8128 potential barcoding SNPs (with *F*_ST_ = 1) across the 90 clades (Table 1). These barcoding SNPs were distributed evenly genome-wide, with no visible clustering of informative mutations for individual lineages (Additional file 1: Fig. S3). Of these SNPs, 7282 (89.6%) were in genic regions, with mutations leading to 4699 non-synonymous (NS) and 2564 synonymous (S) amino acid changes, as well as 20 changes in non-coding genes. By focusing on essential genes, 889 (10.9%) SNPs remained (499 NS, 390 S). Furthermore, variants in drug-resistance-associated genes were removed, leaving 824 SNPs (464 NS and 360 S mutations). Across all lineages, except lineages 8 (*N* = 2) and 9 (*N* = 3) which had small sample sizes, we compared the distribution of gene functions for all potential barcoding SNPs in all characterised genes (7060/7282 SNPs) with only those in essential (and non-drug resistance) loci (790/824 SNPs) (Additional file 1: Fig. S4). The distribution of gene function for all potential barcoding SNPs is similar across all lineages. However, after filtering for essential and non-drug-resistant genes, lineage 2 has a relatively high proportion of non-synonymous SNP mutations in cell wall and cell process genes, whilst for lineage 6, *M. bovis*, *M. caprae* and *M. orygis*, there are relatively higher proportions of non-synonymous SNP mutations in intermediary metabolism and pathway genes. For 11 (sub-)lineages, there were no potential barcoding SNPs lying within essential and non-drug resistance genes, so they were identified in non-essential and non-PE/PPE loci (Additional file 1: Table S3) (180 SNPs, 61 synonymous mutations).

By considering only the SNPs with synonymous changes, similar to the selection strategy applied in [1], a total of 421 SNPs were considered suitable for barcoding the 90 (sub-)lineages (Table 1; Additional file 1: Table S2). Of these, 20 SNPs represented (sub-)lineages in the 62-SNP barcode [1] and were therefore retained, leading to 70 new SNPs chosen for final (sub-)lineage classification (Additional file 1: Table S3). Across the 60 (sub-)lineages common to the 62- and 90-SNP barcodes, the 40 new SNPs had higher *F*_ST_ values than those in the old barcode (Additional file 1: Fig. S5). Using the test set (*N* = 17,395) which had representation of 86 of the 90 (sub-)lineages, we found that the minimal set of 90 SNPs had perfect predictive performance for all clades (all sensitivities and specificities of value 1). This analysis excluded four (sub-)lineages (3.1.2.2, 4.6.2.1, 8 and 9), which had no test samples.

### Comparisons to other software

The barcode was compared to lineage predictions from SNP-IT [30] software, a 27 strain-type system covering MTBC, including 6 animal lineages that are not present in our large dataset. First, we assessed the assigned major MTBC lineages (1–6) by both barcodes and found complete concordance. Second, we quantified how the increased number of strain types in our barcode (*n* = 90) improved the resolution of sub-lineage assignment over the SNP-IT tool. For 14 of the 21 SNP-IT strain types present in our data, the 90-SNP approach provides higher resolution of clades (range 2 to 15 sub-lineages per SNP-IT clade) (Additional file 1: Fig. S6). Six other strain types have direct mapping between our barcode and SNP-IT, and there is one instance where isolates classified as *M. bovis* with our barcode are further classified into *M. bovis BCG* and *M. bovis bovis* using SNP-IT.

## Discussion

MTBC strain types and lineages are distributed phylogeographically and have been associated with differences in the emergence of drug resistance, transmissibility, virulence, host response, vaccine efficacy, disease site and severity [5, 6, 34]. However, further research into lineage, genotype–phenotype associations are required. Such research needs to be underpinned by molecular barcodes of MTBC (sub-)lineages, strain types and species. Here, we updated a 62-SNP barcode that forms a highly resolved phylogenetic identification system that determines 7 lineages, 64 sub-lineages and *M. bovis*, but was constructed using ~ 1600 MTBC isolates with WGS data [1]. Using twenty-fold more MTBC isolates with WGS data, we identified and validated a set of 90 robust SNPs (of 421 alternatives) to cover a global phylogeny of 9 lineages, 87 sub-lineages, *M. bovis*, *M. caprae* and *M. orygis*. These SNPs can be used to construct high-resolution and reproducible phylogenies, which can be incorporated within diagnostic assays and assess genotype–phenotype associations. By extending an established 62-SNP barcode system with a flexible nomenclature [1], it was possible to update and add seamlessly (sub-)lineages and species and in the future include potentially novel strain types should they be reported. Such modifications could involve inclusion of SNPs to barcode other MTBC animal lineages or partitioning of *M. africanum* lineages 5 and 6 into sub-lineages [3]. Further, incorporating drug resistance loci will further enhance the usefulness of the 90-SNP barcode as an important tool for tuberculosis control and elimination activities worldwide. To assist this, the 90-SNP variants have been incorporated into the publicly available *TB-Profiler* informatics tool [14], which predicts resistance to 14 anti-tuberculosis drugs from WGS data.

Our barcode development focused on SNPs, but future work could include other types of strain-specific polymorphisms (e.g. insertions, deletions and large structural variants), which are less common than SNPs, but may have major functional consequences. An analysis of the gene ontologies of the barcoding SNPs revealed some differences across lineages, but there is a need to the characterise functional effects of the lineage-specific SNP variants, as these could provide insights into disease control measures. Overall, we have provided an updated molecular barcode for MTBC strain types, with ninety robust markers that can be detected from applications of WGS or integrated within high-throughput genotyping or sequencing (e.g. amplicon) platforms to inform on-going TB surveillance and control.

## Conclusions

The use of molecular barcoding of MTBC bacteria causing tuberculosis can provide insights into outbreaks and help to reveal strain types that are more virulent and prone to drug resistance. In an analysis of 35,298 isolates from MTBC, we update an established 62-SNP barcode with a minimal set of 90 genetic markers, which now cover *M. tuberculosis* (7 lineages, 85 sub-lineages), *M. africanum* (2 lineages), *M. bovis*, *M. caprae* and *M. orygis* bacteria. The new barcode has been implemented within the publicly available *TB-Profiler* informatics tool, to assist the rapid, simple and reliable phylogenetic identification of individual MTBC isolates, thereby aiding clinical studies in the tracking, maintenance and phenotypic determination of MTBC pathogens.

## Supplementary Information

**Additional file 1: Table S1.** The study samples (*N*=35,298) used and their lineages. **Table S2.** Robust barcoding SNPs (421 SNPs, including the 90 SNPs in **Table S3**). **Table S3.** The ninety minimal barcoding SNPs. **Figure S1.** Population structure of the *Mycobacterium tuberculosis* complex isolates by lineage. **Figure S2.** Examples of discrepancies using the 62-SNP barcode**. Figure S3.** The genome-wide distribution of barcoding SNPs (F_ST_=1) for each lineage. **Figure S4.** Functional differences between genes containing lineage-barcoding (F_ST_=1) SNPs. **Figure S5.** Differentiation of sub-lineages when comparing the 62- versus 90-SNP barcodes. **Figure S6.** The increased resolution of our 90-SNP barcode (implemented in *TB-Profiler* software) over the comparable (sub-)lineages of the *SNP-IT* tool.

## Data Availability

All raw sequence data is available from the EBI short read archive. A dedicated GitHub repository (https://github.com/GaryNapier/tb-lineages) [35] contains the list of accession numbers and code. The new (sub-)lineages have been implemented within the TB-Profiler tool https://github.com/jodyphelan/TBProfiler [14].

## Abbreviations

MDR-TB: Multidrug-resistant TB
MTBC: *Mycobacterium tuberculosis* complex
RD: Region of difference
SNP: Single nucleotide polymorphism
TB: Tuberculosis
WGS: Whole genome sequencing
XDR-TB: Extensively drug-resistant TB
