# Supplementary material for: Robust barcoding and identification of Mycobacterium tuberculosis lineages for epidemiological and clinical studies
Source: Genome Med. 2020 Dec 14;12:114. doi: 10.1186/s13073-020-00817-3 (PMC7734807; doi:10.1186/s13073-020-00817-3)
Supplement: Supplementary file 1 — Additional file 1: Table S1. The study samples (N=35,298) used and their lineages. Table S2. Robust barcoding SNPs (421 SNPs, including the 90 SNPs in Table S3). Table S3. The ninety minimal barcoding SNPs. Figure S1. Population structure of the Mycobacterium tuberculosis complex isolates by lineage. Figure S2. Examples of discrepancies using the 62-SNP barcode. Figure S3. The genome-wide distribution of barcoding SNPs (FST=1) for each lineage. Figure S4. Functional differences between genes containing lineage-barcoding (FST=1) SNPs. Figure S5. Differentiation of sub-lineages when comparing the 62- versus 90-SNP barcodes. Figure S6. The increased resolution of our 90-SNP barcode (implemented in TB-Profiler software) over the comparable (sub-)lineages of the SNP-IT tool. [file 13073_2020_817_MOESM1_ESM.pdf]

Table S1: **The study samples (N=35,298) used and their lineages (L)**

Region	No. countries	L1	L2	L3	L4	L5	L6	L7	L8	L9	*M.bovis, caprae, orygis*
North America	10	317	390	228	1414	3	-	-	-	-	8
South America	6	4	57	1	851	-	-	-	-	-	2
Western Europe	14	749	1047	1880	4551	17	27	-	-	-	178
Eastern Europe	15	3	974	5	788	-	-	-	-	-	2
North Africa	5	-	-	-	60	-	-	-	-	-	-
West Africa	12	5	5	-	184	212	114	-	-	-	1
East Africa	9	43	6	36	112	-	1	64	2	3*	1
South Africa	3	80	1451	145	3380	-	-	-	-	-	1
Central Africa	10	291	93	238	1965	25	2	-	-	-	11
Central Asia	15	40	344	418	191	-	-	-	-	-	3
South Asia	2	309	74	109	55	-	-	-	-	-	-
East Asia	15	1202	3513	50	1060	-	-	-	-	-	-
Oceania	1	6	21	5	33	-	-	-	-	-	-
Unknown	-	316	903	810	3559	24	23	-	-	-	203
Total	118	3365	8878	3925	18203	281	167	64	2	3	410
Training N	-	2162	4556	2654	8320	26	32	38	2	3	110
Test N	-	1203	4322	1271	9883	255	135	26	-	-	300

* predicted in reference [3]

Table S2: Robust barcoding SNPs (421 SNPs, including the 90 minimal barcoding SNPs in Table S3)

Lineage	Position	Change	Strand	Amino acid	Gene	Locus	Functional Category
1	272678	C->T	-	54A	*Rv0227c*	Rv0227c	cell wall and cell processes
1	344288	C->G	+	89S	*eccB3*	Rv0283	cell wall and cell processes
1	615938	G->A	+	368E	*hemL*	Rv0524	IMR
1	646531	A->T	+	78T	*menD*	Rv0555	IMR
1	811492	C->G	+	40V	*rplN*	Rv0714	information pathways
1	812502	C->T	+	148V	*rplE*	Rv0716	information pathways
1	865761	C->T	+	392H	*purD*	Rv0772	IMR
1	1560912	G->A	+	156E	*pyrF*	Rv1385	IMR
1	1590555	C->T	+	53T	*ribA2*	Rv1415	IMR
1	2897528	G->A	-	92A	*aspS*	Rv2572c	information pathways
1	3233605	G->A	-	179L	*ftsY*	Rv2921c	cell wall and cell processes
1	3647591	A->G	-	73N	*rmlD*	Rv3266c	IMR
1	3830566	G->A	-	318S	*guaB2*	Rv3411c	IMR
1	4022652	G->A	-	384S	*cysS1*	Rv3580c	information pathways
1	4081987	G->C	-	245A	*Rv3644c*	Rv3644c	information pathways
1	4081996	G->C	-	242P	*Rv3644c*	Rv3644c	information pathways
1	4155266	C->G	+	509G	*leuA*	Rv3710	IMR
1.1	2989683	C->T	+	131A	*aftC*	Rv2673	cell wall and cell processes
1.1	4404247	G->A	+	352L	*Rv3915*	Rv3915	IMR
1.1.1	529363	C->T	+	252V	*groEL2*	Rv0440	VDA
1.1.1	870112	C->T	+	35A	*purB*	Rv0777	IMR
1.1.1	1261056	C->T	-	97G	*metE*	Rv1133c	IMR
1.1.1	1924765	T->C	+	313L	*pyrG*	Rv1699	IMR
1.1.1	2078024	G->A	+	716P	*gcvB*	Rv1832	IMR
1.1.1.1	1750465	T->C	+	924L	*dnaE1*	Rv1547	information pathways
1.1.1.1	2412584	G->A	-	256A	*murG*	Rv2153c	cell wall and cell processes
1.1.1.1	2994964	C->T	-	33G	*hemE*	Rv2678c	IMR
1.1.2	2622402	G->A	-	17A	*dnaG*	Rv2343c	information pathways
1.1.2	3879882	G->A	-	63N	*rpsM*	Rv3460c	information pathways
1.1.2	4157259	G->A	+	93V	*Rv3712*	Rv3712	IMR
1.1.3	15177	C->G	+	88A	*trpG*	Rv0013	IMR
1.1.3	1491275	G->A	-	346H	*glgB*	Rv1326c	IMR
1.1.3.1	403481	C->T	-	787R	*Rv0338c*	Rv0338c	IMR
1.1.3.1	1345104	G->A	-	22L	*dapD*	Rv1201c	IMR
1.1.3.2	285096	G->T	-	586R	*aftD*	Rv0236c	cell wall and cell processes
1.1.3.2	2369187	C->T	-	181L	*prcA*	Rv2109c	IMR
1.1.3.2	2369460	G->A	-	90D	*prcA*	Rv2109c	IMR
1.1.3.2	2418554	G->A	-	151L	*murF*	Rv2157c	cell wall and cell processes
1.1.3.2	4084405	G->A	+	533R	*Rv3645*	Rv3645	cell wall and cell processes

1.1.3.2	4154816	G->A	+	359P	*leuA*	Rv3710	IMR
1.1.3.3	2738352	C->T	-	445A	*obg*	Rv2440c	IMR
1.1.3.3	3337585	G->A	-	111D	*ddlA*	Rv2981c	cell wall and cell processes
1.2	1136017	A->G	-	155G	*prsA*	Rv1017c	IMR
1.2	1553855	C->T	+	208T	*pyrB*	Rv1380	IMR
1.2.1	590595	G->A	+	171Q	*proC*	Rv0500	IMR
1.2.1	2640960	C->T	-	35A	*glyS*	Rv2357c	information pathways
1.2.1	2847191	G->A	-	714D	*fas*	Rv2524c	lipid metabolism
1.2.1	3387252	G->A	-	260A	*fixB*	Rv3028c	IMR
1.2.1	4402048	C->T	+	107T	*trxB2*	Rv3913	IMR
1.2.2	528781	G->A	+	58E	*groEL2*	Rv0440	VDA
1.2.2	1567985	A->G	+	387E	*metK*	Rv1392	IMR
1.2.2	2639868	A->G	-	399D	*glyS*	Rv2357c	information pathways
1.2.2	2840849	G->C	-	2828G	*fas*	Rv2524c	lipid metabolism
1.2.2	3629612	G->A	-	12T	*sahH*	Rv3248c	IMR
1.2.2	3862181	C->T	-	70Q	*rplM*	Rv3443c	information pathways
1.2.2	4024368	C->T	-	224L	*ispD*	Rv3582c	IMR
1.2.2	4237383	C->A	+	73T	*dprE2*	Rv3791	lipid metabolism
1.2.2.1	2737201	A->C	-	349S	*proB*	Rv2439c	IMR
1.3	2763624	G->A	-	51L	*clpP1*	Rv2461c	IMR
1.3	4238120	G->A	+	63Q	*aftA*	Rv3792	cell wall and cell processes
1.3.1	1245275	C->T	+	49A	*gnd2*	Rv1122	IMR
1.3.1	1651063	C->G	+	116R	*Rv1463*	Rv1463	cell wall and cell processes
1.3.1	3323665	C->A	-	13P	*Rv2968c*	Rv2968c	cell wall and cell processes
1.3.1	3787156	G->A	+	281Q	*otsB2*	Rv3372	VDA
1.3.2	61842	T->C	+	483L	*dnaB*	Rv0058	information pathways
1.3.2	1492049	C->T	-	88L	*glgB*	Rv1326c	IMR
1.3.2	3147316	G->A	-	186G	*infB*	Rv2839c	information pathways
1.3.2	4200993	T->G	+	191A	*tyrA*	Rv3754	IMR
2	282892	C->T	-	1320T	*aftD*	Rv0236c	cell wall and cell processes
2	811753	C->T	+	4H	*rplX*	Rv0715	information pathways
2	4254431	G->A	-	506D	*accD4*	Rv3799c	lipid metabolism
2	4308395	G->A	-	174L	*serS*	Rv3834c	information pathways
2.1	648465	A->G	+	169A	*Rv0556*	Rv0556	cell wall and cell processes
2.1	1135798	C->T	-	228L	*prsA*	Rv1017c	IMR
2.1	2737453	A->C	-	265R	*proB*	Rv2439c	IMR
2.1	4165481	G->C	-	417P	*dnaZX*	Rv3721c	information pathways
2.2	195682	C->G	+	230V	*fadD5*	Rv0166	lipid metabolism
2.2	363464	G->A	+	71R	*Rv0298*	Rv0298	conserved hypotheticals
2.2	465300	C->T	+	630F	*Rv0386*	Rv0386	regulatory proteins
2.2	892416	C->T	-	286V	*Rv0799c*	Rv0799c	conserved hypotheticals

2.2	1288698	G->A	+	457G	*narG*	Rv1161	IMR
2.2	1695037	G->A	-	36F	*Rv1504c*	Rv1504c	conserved hypotheticals
2.2	1849051	C->T	-	995P	*lysX*	Rv1640c	information pathways
2.2	2112832	A->C	-	45A	*Rv1865c*	Rv1865c	IMR
2.2	2202500	C->T	+	121H	*higA*	Rv1956	VDA
2.2	2505085	G->A	-	205A	*cobC*	Rv2231c	IMR
2.2	2775361	C->T	+	30R	*Rv2472*	Rv2472	conserved hypotheticals
2.2	2903439	G->A	-	31S	*Rv2578c*	Rv2578c	conserved hypotheticals
2.2	3477942	A->G	+	98T	*moaA1*	Rv3109	IMR
2.2	3587446	G->A	-	32L	*Rv3210c*	Rv3210c	conserved hypotheticals
2.2	4050811	G->A	-	691Y	*ftsH*	Rv3610c	cell wall and cell processes
2.2	4186678	G->A	+	15L	*Rv3736*	Rv3736	regulatory proteins
2.2	4189210	G->T	+	504P	*Rv3737*	Rv3737	cell wall and cell processes
2.2.1	2078246	C->G	+	790G	*gcvB*	Rv1832	IMR
2.2.1	4158493	C->T	+	89I	*cobQ2*	Rv3713	IMR
2.2.1.1	1947282	A->G	-	46R	*vapC12*	Rv1720c	VDA
2.2.1.1	4080525	C->T	-	12R	*fic*	Rv3641c	cell wall and cell processes
2.2.1.2	1692069	A->G	+	60A	*Rv1501*	Rv1501	conserved hypotheticals
2.2.2	346693	G->T	+	353S	*eccC3*	Rv0284	cell wall and cell processes
2.2.2	1565566	C->T	+	42P	*dfp*	Rv1391	IMR
2.2.2	2640807	G->A	-	86V	*glyS*	Rv2357c	information pathways
2.2.2	3147511	T->G	-	121A	*infB*	Rv2839c	information pathways
3	342873	C->T	+	248V	*eccA3*	Rv0282	cell wall and cell processes
3	652950	T->C	+	60R	*grcC1*	Rv0562	IMR
3	1450316	C->T	+	314A	*thrA*	Rv1294	IMR
3	1764225	C->T	+	266A	*ilvA*	Rv1559	IMR
3	1925136	G->A	+	436V	*pyrG*	Rv1699	IMR
3	2738221	G->A	-	9I	*proB*	Rv2439c	IMR
3	2782498	G->A	-	515D	*Rv2477c*	Rv2477c	cell wall and cell processes
3	4396495	C->A	+	768G	*Rv3909*	Rv3909	conserved hypotheticals
3.1	958362	C->G	+	690A	*fadB*	Rv0860	lipid metabolism
3.1.1	1591545	G->T	+	383P	*ribA2*	Rv1415	IMR
3.1.1	3023684	G->A	+	40T	*ideR*	Rv2711	regulatory proteins
3.1.2	1914217	C->A	+	206R	*tyrS*	Rv1689	information pathways
3.1.2	3722702	G->C	-	310L	*trpS*	Rv3336c	information pathways
3.1.2.1	1237818	C->G	-	125L	*Rv1111c*	Rv1111c	conserved hypotheticals
3.1.2.1	2020120	C->T	+	288T	*eccC5*	Rv1783	cell wall and cell processes
3.1.2.1	2185538	G->A	-	217V	*fadE17*	Rv1934c	lipid metabolism
3.1.2.1	2245499	C->T	+	97S	*Rv2000*	Rv2000	conserved hypotheticals
3.1.2.1	2271143	G->A	-	202V	*Rv2025c*	Rv2025c	cell wall and cell processes
3.1.2.1	3557911	C->T	-	145L	*Rv3191c*	Rv3191c	insertion seqs and phages

3.1.2.1	4053713	C->G	-	45R	*Rv3612c*	Rv3612c	conserved hypotheticals
3.1.2.2	343092	C->T	+	321F	*eccA3*	Rv0282	cell wall and cell processes
3.1.2.2	346898	C->T	+	422L	*eccC3*	Rv0284	cell wall and cell processes
3.1.2.2	1862121	C->T	+	788H	*pheT*	Rv1650	information pathways
3.1.2.2	2415042	G->T	-	451P	*murD*	Rv2155c	cell wall and cell processes
3.1.2.2	2874344	G->A	-	714R	*alaS*	Rv2555c	information pathways
3.1.2.2	3629291	A->G	-	119G	*sahH*	Rv3248c	IMR
3.2	17842	G->C	-	307A	*pknA*	Rv0015c	regulatory proteins
3.2	1919627	G->A	+	294V	*ppnK*	Rv1695	IMR
4	2825466	A->G	+	263K	*Rv2509*	Rv2509	IMR
4	2994187	C->T	-	292L	*hemE*	Rv2678c	IMR
4	3830695	G->A	-	275A	*guaB2*	Rv3411c	IMR
4.1	62657	G->A	+	754P	*dnaB*	Rv0058	information pathways
4.1	284623	G->A	-	743T	*aftD*	Rv0236c	cell wall and cell processes
4.1	902413	C->T	+	101V	*purF*	Rv0808	IMR
4.1.1	265968	C->G	+	154R	*echA1*	Rv0222	lipid metabolism
4.1.1	514245	C->T	-	359V	*ctpH*	Rv0425c	cell wall and cell processes
4.1.1	869440	C->T	-	108L	*Rv0776c*	Rv0776c	conserved hypotheticals
4.1.1	1256806	C->T	+	225D	*prpC*	Rv1131	IMR
4.1.1	1952601	C->T	+	250H	*Rv1726*	Rv1726	IMR
4.1.1	2158582	G->A	-	170G	*fadB5*	Rv1912c	lipid metabolism
4.1.1	2603797	G->A	-	142I	*lppP*	Rv2330c	cell wall and cell processes
4.1.1	2752854	G->A	-	47D	*Rv2452c*	Rv2452c	conserved hypotheticals
4.1.1	3129359	C->T	-	805K	*Rv2823c*	Rv2823c	conserved hypotheticals
4.1.1	3231091	C->A	-	472V	*amt*	Rv2920c	cell wall and cell processes
4.1.1	3597737	C->T	-	10V	*TB7.3*	Rv3221c	lipid metabolism
4.1.1	4003130	G->A	+	498V	*fadD3*	Rv3561	lipid metabolism
4.1.1	4306767	G->A	-	15G	*Rv3832c*	Rv3832c	conserved hypotheticals
4.1.1.1	1006080	G->A	-	161V	*prrA*	Rv0903c	regulatory proteins
4.1.1.1	2824839	C->T	+	54A	*Rv2509*	Rv2509	IMR
4.1.1.2	1109535	G->A	+	88K	*galU*	Rv0993	IMR
4.1.1.2	3213615	G->A	-	80Y	*lepB*	Rv2903c	cell wall and cell processes
4.1.1.3	896356	C->T	+	179T	*purL*	Rv0803	IMR
4.1.1.3	4154051	G->A	+	104R	*leuA*	Rv3710	IMR
4.1.1.3	4229087	C->T	+	247N	*glfT1*	Rv3782	cell wall and cell processes
4.1.1.3.1	286300	C->A	-	184A	*aftD*	Rv0236c	cell wall and cell processes
4.1.1.3.1	2739087	C->T	-	200V	*obg*	Rv2440c	IMR
4.1.1.3.1	4269540	G->A	-	98P	*ubiA*	Rv3806c	cell wall and cell processes
4.1.2	3147742	A->G	-	44V	*infB*	Rv2839c	information pathways
4.1.2.1	342340	C->T	+	71L	*eccA3*	Rv0282	cell wall and cell processes
4.1.2.1	4256758	G->C	-	1463P	*pks13*	Rv3800c	lipid metabolism

4.1.2.1	4331585	G->A	-	1499D	*gltB*	Rv3859c	IMR
4.1.2.1.1	2488724	C->G	+	370P	*glnA1*	Rv2220	IMR
4.1.2.1.1	2640369	G->A	-	232Y	*glyS*	Rv2357c	information pathways
4.1.2.1.1	4404313	G->A	+	374L	*Rv3915*	Rv3915	IMR
4.1.3	1564799	C->T	+	133P	*gmk*	Rv1389	IMR
4.1.3	2450245	G->A	-	302A	*pimB*	Rv2188c	lipid metabolism
4.1.3	4228101	C->T	+	191D	*rfbE*	Rv3781	cell wall and cell processes
4.1.4	58786	G->C	+	67V	*ssb*	Rv0054	information pathways
4.1.4	590250	G->T	+	56T	*proC*	Rv0500	IMR
4.1.4	2844014	G->A	-	1773F	*fas*	Rv2524c	lipid metabolism
4.1.4	4391663	G->A	-	471L	*pcnA*	Rv3907c	information pathways
4.2	1466779	C->T	+	313I	*atpD*	Rv1310	IMR
4.2	1568018	C->T	+	398D	*metK*	Rv1392	IMR
4.2	1670814	C->T	+	134G	*Rv1480*	Rv1480	conserved hypotheticals
4.2	1872211	G->A	+	283A	*argG*	Rv1658	IMR
4.2	2748087	G->A	-	713S	*valS*	Rv2448c	information pathways
4.2	3198496	G->A	-	204I	*tsf*	Rv2889c	information pathways
4.2	3469694	G->T	-	30A	*smpB*	Rv3100c	VDA
4.2	3666905	C->T	+	183T	*accA3*	Rv3285	lipid metabolism
4.2.1	783601	A->C	+	373R	*fusA1*	Rv0684	information pathways
4.2.1	3646964	C->G	-	282L	*rmlD*	Rv3266c	IMR
4.2.1.1	870187	C->T	+	60D	*purB*	Rv0777	IMR
4.2.1.1	1652216	A->G	+	233K	*csd*	Rv1464	IMR
4.2.2	353766	T->C	+	228I	*eccD3*	Rv0290	cell wall and cell processes
4.2.2	2420503	A->G	-	36L	*murE*	Rv2158c	cell wall and cell processes
4.2.2.1	1131	C->A	+	377I	*dnaA*	Rv0001	information pathways
4.2.2.1	1455780	T->C	+	96L	*prfA*	Rv1299	information pathways
4.2.2.2	611463	G->A	-	204T	*Rv0519c*	Rv0519c	cell wall and cell processes
4.2.2.2	1233285	G->A	-	224Y	*Rv1106c*	Rv1106c	IMR
4.2.2.2	1880850	G->A	+	1849L	*pks7*	Rv1661	lipid metabolism
4.2.2.2	2153246	T->G	-	213R	*Rv1907c*	Rv1907c	conserved hypotheticals
4.2.2.2	2156847	G->A	-	151T	*Rv1910c*	Rv1910c	cell wall and cell processes
4.2.2.2	2923264	G->A	-	324L	*ruvB*	Rv2592c	information pathways
4.2.2.2	3141827	C->G	-	132L	*ugpA*	Rv2835c	cell wall and cell processes
4.2.2.2	3416734	G->A	+	10L	*dinP*	Rv3056	information pathways
4.2.2.2	3497369	G->A	+	273L	*Rv3131*	Rv3131	conserved hypotheticals
4.2.2.2	4298106	C->A	-	500S	*pks2*	Rv3825c	lipid metabolism
4.3	1452071	C->A	+	25G	*thrB*	Rv1296	IMR
4.3	3191027	G->A	-	199L	*cdsA*	Rv2881c	lipid metabolism
4.3.1	825585	T->C	+	262Y	*secY*	Rv0732	cell wall and cell processes
4.3.1.1	1647807	T->C	+	273I	*Rv1461*	Rv1461	conserved hypotheticals

4.3.1.1	3360032	G->A	-	185T	*ilvC*	Rv3001c	IMR
4.3.2	3414791	G->C	-	56A	*nrdH*	Rv3053c	information pathways
4.3.2.1	784581	G->C	+	699T	*fusA1*	Rv0684	information pathways
4.3.2.1	1451542	C->T	+	282A	*thrC*	Rv1295	IMR
4.3.2.1	1592015	C->T	+	115G	*ribH*	Rv1416	IMR
4.3.2.1	2844689	G->C	-	1548L	*fas*	Rv2524c	lipid metabolism
4.3.3	2077253	G->A	+	459T	*gcvB*	Rv1832	IMR
4.3.4	1297327	G->A	+	392V	*lpqW*	Rv1166	cell wall and cell processes
4.3.4.1	1274335	G->A	+	327L	*mmpL13b*	Rv1146	cell wall and cell processes
4.3.4.1	2199684	G->A	-	117F	*Rv1949c*	Rv1949c	conserved hypotheticals
4.3.4.1	3244674	G->A	+	326R	*fadD26*	Rv2930	lipid metabolism
4.3.4.1	3285945	C->G	+	292A	*mmpL7*	Rv2942	cell wall and cell processes
4.3.4.2	784440	G->T	+	652A	*fusA1*	Rv0684	information pathways
4.3.4.2.1	225495	T->C	-	359V	*Rv0193c*	Rv0193c	conserved hypotheticals
4.4	4238963	C->T	+	344H	*aftA*	Rv3792	cell wall and cell processes
4.4	4307886	G->A	-	343R	*serS*	Rv3834c	information pathways
4.4.1	2905505	G->A	-	196T	*hisS*	Rv2580c	information pathways
4.4.1	3147376	G->A	-	166P	*infB*	Rv2839c	information pathways
4.4.1	3664135	G->A	+	149R	*accE5*	Rv3281	lipid metabolism
4.4.1	3813473	G->A	-	202L	*guaA*	Rv3396c	IMR
4.4.1.1	355181	G->A	+	228K	*mycP3*	Rv0291	IMR
4.4.1.1.1	15036	C->G	+	41A	*trpG*	Rv0013	IMR
4.4.1.1.1	1126895	G->A	-	37L	*metS*	Rv1007c	information pathways
4.4.1.1.1	1221479	C->T	+	302V	*glyA1*	Rv1093	IMR
4.4.1.2	342201	C->G	+	24P	*eccA3*	Rv0282	cell wall and cell processes
4.4.1.2	345697	C->T	+	21T	*eccC3*	Rv0284	cell wall and cell processes
4.4.1.2	1297981	G->C	+	610V	*lpqW*	Rv1166	cell wall and cell processes
4.4.1.2	1494231	G->A	-	65L	*glgE*	Rv1327c	IMR
4.4.1.2	1803959	G->T	+	222G	*hisA*	Rv1603	IMR
4.4.1.2	1808124	A->C	+	74P	*trpE*	Rv1609	IMR
4.4.1.2	2410938	A->T	-	395A	*murC*	Rv2152c	cell wall and cell processes
4.4.1.2	3349093	G->C	-	395P	*gltS*	Rv2992c	information pathways
4.4.1.2	4392120	G->A	-	318H	*pcnA*	Rv3907c	information pathways
4.4.2	985287	G->A	+	495P	*fprB*	Rv0886	IMR
4.4.2	2913091	C->T	-	307V	*secF*	Rv2586c	cell wall and cell processes
4.5	620029	C->T	+	47L	*ccsA*	Rv0529	IMR
4.6	18091	G->A	-	224T	*pknA*	Rv0015c	regulatory proteins
4.6.1	435708	G->A	-	354T	*purA*	Rv0357c	IMR
4.6.1	2440953	G->T	-	256R	*aroG*	Rv2178c	IMR
4.6.1	4260268	G->C	-	293A	*pks13*	Rv3800c	lipid metabolism
4.6.1.1	4406749	G->A	-	261L	*parA*	Rv3918c	cell wall and cell processes

4.6.1.2	1098698	C->G	+	397G	*mprB*	Rv0982	regulatory proteins
4.6.2	4260742	G->A	-	135P	*pks13*	Rv3800c	lipid metabolism
4.6.2.1	896119	C->T	+	100F	*purL*	Rv0803	IMR
4.6.2.1	2897684	A->G	-	40D	*aspS*	Rv2572c	information pathways
4.6.2.2	118469	G->A	+	252A	*Rv0102*	Rv0102	cell wall and cell processes
4.6.2.2	1352350	C->T	+	69V	*gpgS*	Rv1208	IMR
4.6.2.2	2369118	G->A	-	204G	*prcA*	Rv2109c	IMR
4.6.2.2	2875883	C->T	-	201L	*alaS*	Rv2555c	information pathways
4.6.2.2	3354625	G->A	-	149L	*serA1*	Rv2996c	IMR
4.6.2.2	3360152	C->A	-	145P	*ilvC*	Rv3001c	IMR
4.6.3	734562	G->A	+	103K	*nusG*	Rv0639	information pathways
4.6.3	2516158	C->G	+	285T	*Rv2242*	Rv2242	conserved hypotheticals
4.6.3	2516365	T->C	+	354Y	*Rv2242*	Rv2242	conserved hypotheticals
4.6.4	4236903	G->A	+	375A	*dprE1*	Rv3790	lipid metabolism
4.6.5	17665	G->A	-	366N	*pknA*	Rv0015c	regulatory proteins
4.6.5	1553876	C->G	+	215A	*pyrB*	Rv1380	IMR
4.7	716918	G->A	+	85T	*vapC30*	Rv0624	VDA
4.7	3270289	C->A	+	851R	*ppsE*	Rv2935	lipid metabolism
4.7	4112595	G->A	-	307A	*Rv3671c*	Rv3671c	IMR
4.8	1130526	G->A	+	112A	*ispE*	Rv1011	IMR
4.8.1	2914906	G->C	-	277T	*secD*	Rv2587c	cell wall and cell processes
4.8.1	3348870	G->A	-	470L	*gltS*	Rv2992c	information pathways
4.8.1	3389922	T->G	+	274P	*Rv3030*	Rv3030	conserved hypotheticals
4.8.2	2417281	G->A	-	65Y	*murX*	Rv2156c	cell wall and cell processes
4.8.2	3404883	G->A	-	13A	*ctaD*	Rv3043c	IMR
4.8.3	616408	C->G	+	62A	*Rv0525*	Rv0525	conserved hypotheticals
4.9	420008	G->A	+	58A	*dnaK*	Rv0350	VDA
4.9	903913	C->T	+	63G	*purM*	Rv0809	IMR
4.9	3367765	A->G	-	343G	*gatB*	Rv3009c	information pathways
4.9.1	119600	C->G	+	629V	*Rv0102*	Rv0102	cell wall and cell processes
4.9.1	1940611	G->C	+	108A	*engA*	Rv1713	IMR
4.9.1	4165205	C->T	-	509A	*dnaZX*	Rv3721c	information pathways
5	345317	G->C	+	432V	*eccB3*	Rv0283	cell wall and cell processes
5	352646	G->A	+	166P	*espG3*	Rv0289	cell wall and cell processes
5	801959	C->T	+	166R	*rplD*	Rv0702	information pathways
5	1505806	C->T	+	244P	*murI*	Rv1338	cell wall and cell processes
5	1555432	C->T	+	415T	*pyrC*	Rv1381	IMR
5	1578212	A->C	+	200A	*priA*	Rv1402	information pathways
5	1649265	G->A	+	759L	*Rv1461*	Rv1461	conserved hypotheticals
5	1799921	C->A	+	113G	*hisD*	Rv1599	IMR
5	2485956	G->A	+	228E	*lipA*	Rv2218	IMR

5	2859147	C->T	-	48K	*Efp*	Rv2534c	Information pathways
5	3882025	G->A	+	63L	*rmlB*	Rv3464	IMR
5	4086604	G->A	-	218Y	*topA*	Rv3646c	information pathways
5	4387392	G->A	-	168F	*Rv3902c*	Rv3902c	conserved hypotheticals
6	982363	G->T	-	64G	*serC*	Rv0884c	IMR
6	1069146	C->T	+	314G	*purH*	Rv0957	IMR
6	1372002	C->T	-	316L	*mrp*	Rv1229c	IMR
6	1811964	C->A	+	280R	*trpB*	Rv1612	IMR
6	1867707	C->T	+	359P	*argJ*	Rv1653	IMR
6	2847737	G->A	-	532I	*fas*	Rv2524c	lipid metabolism
6	3213255	C->T	-	200K	*lepB*	Rv2903c	cell wall and cell processes
6	3862148	G->A	-	81P	*rplM*	Rv3443c	information pathways
6	4086697	G->T	-	187A	*topA*	Rv3646c	information pathways
6	4236891	C->A	+	371P	*dprE1*	Rv3790	lipid metabolism
7	349081	G->A	+	1149L	*eccC3*	Rv0284	cell wall and cell processes
7	784143	A->C	+	553A	*fusA1*	Rv0684	information pathways
7	811642	C->T	+	90D	*rplN*	Rv0714	information pathways
7	896431	G->A	+	204L	*purL*	Rv0803	IMR
7	1125894	A->C	-	370L	*metS*	Rv1007c	information pathways
7	1137518	G->A	-	181N	*glmU*	Rv1018c	cell wall and cell processes
7	1297084	G->A	+	311L	*lpqW*	Rv1166	cell wall and cell processes
7	1365895	G->A	+	7T	*htrA*	Rv1223	IMR
7	1463776	C->T	+	183V	*atpA*	Rv1308	IMR
7	1561245	C->T	+	267A	*pyrF*	Rv1385	IMR
7	1663221	T->G	-	942S	*acn*	Rv1475c	IMR
7	1799774	C->T	+	64A	*hisD*	Rv1599	IMR
7	1867937	C->T	+	32V	*argB*	Rv1654	IMR
7	2086202	C->G	-	260V	*glcB*	Rv1837c	IMR
7	2380244	G->A	-	139A	*hisG*	Rv2121c	IMR
7	2406193	G->C	-	217L	*Rv2147c*	Rv2147c	conserved hypotheticals
7	2621157	T->C	-	432A	*dnaG*	Rv2343c	information pathways
7	2759363	A->G	-	42C	*clpX*	Rv2457c	IMR
7	2842238	C->A	-	2365A	*fas*	Rv2524c	lipid metabolism
7	2999030	G->A	-	313G	*dxs1*	Rv2682c	IMR
7	3182040	C->T	-	324A	*dxr*	Rv2870c	IMR
7	3225566	C->T	-	240A	*ffh*	Rv2916c	cell wall and cell processes
7	3324231	G->C	-	82S	*Rv2969c*	Rv2969c	cell wall and cell processes
7	3360233	G->A	-	118A	*ilvC*	Rv3001c	IMR
7	3473482	C->T	-	141R	*prfB*	Rv3105c	information pathways
7	3603631	G->T	+	85G	*aroA*	Rv3227	IMR
7	3635935	C->G	-	111R	*manA*	Rv3255c	IMR

7	3666497	C->T	+	47A	*accA3*	Rv3285	lipid metabolism
7	3667883	C->A	+	509V	*accA3*	Rv3285	lipid metabolism
7	3837064	G->A	-	75G	*groES*	Rv3418c	VDA
7	3860696	G->A	-	225D	*mrsA*	Rv3441c	IMR
7	3878040	A->G	-	156G	*rpoA*	Rv3457c	information pathways
7	4022163	C->T	-	77L	*Rv3579c*	Rv3579c	IMR
7	4086748	C->G	-	170L	*topA*	Rv3646c	information pathways
7	4262608	G->A	-	153I	*fadD32*	Rv3801c	lipid metabolism
7	4267649	A->G	-	396G	*aftB*	Rv3805c	cell wall and cell processes
7	4308411	T->G	-	168L	*serS*	Rv3834c	information pathways
7	4331184	G->A	-	108L	*gltD*	Rv3858c	IMR
8	221190	G->T	-	178V	*ilvD*	Rv0189c	IMR
8	270362	C->A	-	401V	*Rv0226c*	Rv0226c	cell wall and cell processes
8	343314	C->T	+	395A	*eccA3*	Rv0282	cell wall and cell processes
8	344414	G->C	+	131S	*eccB3*	Rv0283	cell wall and cell processes
8	347977	A->C	+	781P	*eccC3*	Rv0284	cell wall and cell processes
8	442072	C->A	-	76A	*fba*	Rv0363c	IMR
8	742270	G->T	-	116S	*Rv0647c*	Rv0647c	conserved hypotheticals
8	896152	C->T	+	111V	*purL*	Rv0803	IMR
8	903262	C->A	+	384V	*purF*	Rv0808	IMR
8	1227518	G->A	-	16T	*fum*	Rv1098c	IMR
8	1260255	C->T	-	364A	*metE*	Rv1133c	IMR
8	1446846	C->T	+	156T	*argS*	Rv1292	information pathways
8	1463923	C->T	+	232T	*atpA*	Rv1308	IMR
8	1505182	C->T	+	36V	*murI*	Rv1338	cell wall and cell processes
8	1553399	C->T	+	56T	*pyrB*	Rv1380	IMR
8	1564640	C->A	+	80L	*gmk*	Rv1389	IMR
8	1629090	C->G	-	370P	*tkt*	Rv1449c	IMR
8	1665285	C->A	-	254P	*acn*	Rv1475c	IMR
8	1805152	C->T	+	100V	*hisF*	Rv1605	IMR
8	1858921	C->G	+	63V	*pheS*	Rv1649	information pathways
8	1914246	C->T	+	215T	*tyrS*	Rv1689	information pathways
8	1922231	C->T	+	230V	*Rv1697*	Rv1697	conserved hypotheticals
8	1941256	C->A	+	323V	*engA*	Rv1713	IMR
8	2086736	G->A	-	82R	*glcB*	Rv1837c	IMR
8	2415402	C->T	-	331K	*murD*	Rv2155c	cell wall and cell processes
8	2440802	A->G	-	307L	*aroG*	Rv2178c	IMR
8	2450263	C->T	-	296V	*pimB*	Rv2188c	lipid metabolism
8	2466760	G->T	+	588T	*asnB*	Rv2201	IMR
8	2862497	G->T	-	60A	*aroB*	Rv2538c	IMR
8	2939600	C->A	-	121L	*Rv2611c*	Rv2611c	lipid metabolism

8	2940079	C->A	-	178S	*pgsA1*	Rv2612c	lipid metabolism
8	3151706	C->T	-	415L	*proS*	Rv2845c	information pathways
8	3333720	C->A	-	23V	*thiL*	Rv2977c	IMR
8	3361394	G->A	-	531A	*ilvB1*	Rv3003c	IMR
8	3362522	G->A	-	155I	*ilvB1*	Rv3003c	IMR
8	3408464	G->A	-	305S	*nrdF2*	Rv3048c	information pathways
8	3469397	G->A	-	129G	*smpB*	Rv3100c	VDA
8	3645324	G->A	-	218S	*manB*	Rv3264c	cell wall and cell processes
8	3786517	G->A	+	68S	*otsB2*	Rv3372	VDA
8	3830815	G->A	-	235D	*guaB2*	Rv3411c	IMR
8	4166096	G->A	-	212S	*dnaZX*	Rv3721c	information pathways
8	4227348	C->T	+	120R	*Rv3780*	Rv3780	conserved hypotheticals
8	4406599	G->A	-	311S	*parA*	Rv3918c	cell wall and cell processes
9	2750052	G->A	-	58T	*valS*	Rv2448c	information pathways
9	3094577	G->A	-	108L	*ribF*	Rv2786c	IMR
9	3370805	G->C	-	210S	*gatA*	Rv3011c	information pathways
9	3414553	G->A	-	44Y	*nrdI*	Rv3052c	information pathways
9	3855303	G->A	-	529I	*glmS*	Rv3436c	IMR
M.bovis	62768	A->G	+	791G	*dnaB*	Rv0058	information pathways
M.bovis	3371401	G->A	-	12L	*gatA*	Rv3011c	information pathways
M.bovis	4229470	T->C	+	71Y	*rfbD*	Rv3783	cell wall and cell processes
M.caprae	904090	T->C	+	122G	*purM*	Rv0809	IMR
M.caprae	918685	C->T	-	22L	*desA1*	Rv0824c	lipid metabolism
M.caprae	1069305	G->C	+	367L	*purH*	Rv0957	IMR
M.caprae	2990241	G->T	+	317S	*aftC*	Rv2673	cell wall and cell processes
M.caprae	3094181	G->A	-	240F	*ribF*	Rv2786c	IMR
M.orygis	44812	G->T	+	417P	*leuS*	Rv0041	information pathways
M.orygis	59181	C->T	+	20C	*rpsR1*	Rv0055	information pathways
M.orygis	268953	C->T	+	97V	*Rv0225*	Rv0225	cell wall and cell processes
M.orygis	500710	G->A	-	103P	*thiE*	Rv0414c	IMR
M.orygis	748320	G->A	+	15A	*rplJ*	Rv0651	information pathways
M.orygis	1125468	C->T	-	512P	*metS*	Rv1007c	information pathways
M.orygis	1236745	G->C	+	187S	*lytB2*	Rv1110	cell wall and cell processes
M.orygis	1344807	G->A	-	121P	*dapD*	Rv1201c	IMR
M.orygis	1449038	C->T	+	337G	*lysA*	Rv1293	IMR
M.orygis	1555342	C->T	+	385A	*pyrC*	Rv1381	IMR
M.orygis	1579197	C->T	+	529L	*priA*	Rv1402	information pathways
M.orygis	1652839	G->C	+	24G	*Rv1465*	Rv1465	IMR
M.orygis	1810542	G->A	+	101S	*trpC*	Rv1611	IMR
M.orygis	2069383	T->C	+	102L	*pgsA2*	Rv1822	lipid metabolism
M.orygis	2076692	G->A	+	272R	*gcvB*	Rv1832	IMR

M.orygis	2078567	T->C	+	897Y	*gcvB*	Rv1832	IMR
M.orygis	2475305	G->A	-	222N	*ilvE*	Rv2210c	IMR
M.orygis	3039261	T->C	-	180A	*dapF*	Rv2726c	IMR
M.orygis	3061003	A->G	-	502L	*ftsK*	Rv2748c	cell wall and cell processes
M.orygis	3147196	G->A	-	226D	*infB*	Rv2839c	information pathways
M.orygis	3148454	G->A	-	325G	*nusA*	Rv2841c	information pathways
M.orygis	3241414	G->A	-	182A	*Rv2927c*	Rv2927c	conserved hypotheticals
M.orygis	3337480	A->G	-	146S	*ddlA*	Rv2981c	cell wall and cell processes
M.orygis	3337675	G->A	-	81G	*ddlA*	Rv2981c	cell wall and cell processes
M.orygis	3667352	C->T	+	332D	*accA3*	Rv3285	lipid metabolism
M.orygis	3770449	G->A	-	67R	*folD*	Rv3356c	IMR
M.orygis	4039853	C->T	-	284L	*clpC1*	Rv3596c	IMR
M.orygis	4311950	T->C	-	240E	*pheA*	Rv3838c	IMR

IMR = Intermediary metabolism and respiration; VDA = virulence, detoxification, adaptation

Table S3: The ninety minimal barcoding SNPs

Lineage	Position	Change	Amino Acid	Gene*	Locus	Functional Category
1	615938	G->A	368E	*hemL*	Rv0524	IMR
1.1	4404247	G->A	352L	*Rv3915*	Rv3915	IMR
1.1.1	**529363**	C->T	252V	*groEL2*	Rv0440	VDA
1.1.1.1	**1750465**	T->C	924L	*dnaE1*	Rv1547	information pathways
1.1.2	2622402	G->A	17A	*dnaG*	Rv2343c	information pathways
1.1.3	1491275	G->A	346H	*glgB*	Rv1326c	IMR
1.1.3.1	**403481**	C->T	787R	*Rv0338c*	Rv0338c	IMR
1.1.3.2	**285096**	G->T	586R	*aftD*	Rv0236c	cell wall and cell processes
1.1.3.3	**2738352**	C->T	445A	*obg*	Rv2440c	IMR
1.2	**1136017**	A->G	155G	*prsA*	Rv1017c	IMR
1.2.1	**590595**	G->A	171Q	*proC*	Rv0500	IMR
1.2.2	**528781**	G->A	58E	*groEL2*	Rv0440	VDA
1.2.2.1	**2737201**	A->C	349S	*proB*	Rv2439c	IMR
1.3	**2763624**	G->A	51L	*clpP1*	Rv2461c	IMR
1.3.1	**1245275**	C->T	49A	*gnd2*	Rv1122	IMR
1.3.2	**61842**	T->C	483L	*dnaB*	Rv0058	information pathways
2	**282892**	C->T	1320T	*aftD*	Rv0236c	cell wall and cell processes
2.1	**648465**	A->G	169A	*Rv0556*	Rv0556	cell wall and cell processes
2.2	2505085	G->A	205A	*cobC**	Rv2231c	IMR
2.2.1	**2078246**	C->G	790G	*gcvB*	Rv1832	IMR
2.2.1.1	**1947282**	A->G	46R	*vapC12**	Rv1720c	VDA
2.2.1.2	**1692069**	A->G	60A	*Rv1501**	Rv1501	conserved hypotheticals
2.2.2	346693	G->T	353S	*eccC3*	Rv0284	cell wall and cell processes
3	**342873**	C->T	248V	*eccA3*	Rv0282	cell wall and cell processes
3.1	**958362**	C->G	690A	*fadB**	Rv0860	lipid metabolism
3.1.1	**1591545**	G->T	383P	*ribA2*	Rv1415	IMR
3.1.2	3722702	G->C	310L	*trpS*	Rv3336c	information pathways
3.1.2.1	1237818	C->G	125L	*Rv1111c**	Rv1111c	conserved hypotheticals
3.1.2.2	2874344	G->A	714R	*alaS*	Rv2555c	information pathways
3.2	**17842**	G->C	307A	*pknA*	Rv0015c	regulatory proteins
4	**2825466**	G->A	263K	*Rv2509*	Rv2509	IMR
4.1	62657	G->A	754P	*dnaB*	Rv0058	information pathways
4.1.1	514245	C->T	359V	*ctpH**	Rv0425c	cell wall and cell processes
4.1.1.1	**1006080**	G->A	161V	*prrA*	Rv0903c	regulatory proteins
4.1.1.2	**1109535**	G->A	88K	*galU*	Rv0993	IMR
4.1.1.3	4229087	C->T	247N	*glfT1*	Rv3782	cell wall and cell processes
4.1.1.3.1	**286300**	C->A	184A	*aftD*	Rv0236c	cell wall and cell processes
4.1.2	**3147742**	A->G	44V	*infB*	Rv2839c	information pathways
4.1.2.1	**342340**	C->T	71L	*eccA3*	Rv0282	cell wall and cell processes
4.1.2.1.1	**2488724**	C->G	370P	*glnA1*	Rv2220	IMR
4.1.3	**1564799**	C->T	133P	*gmk*	Rv1389	IMR

4.1.4	**58786**	G->C	67V	*ssb*	Rv0054	information pathways
4.2	**1466779**	C->T	313I	*atpD*	Rv1310	IMR
4.2.1	783601	A->C	373R	*fusA1*	Rv0684	information pathways
4.2.1.1	**870187**	C->T	60D	*purB*	Rv0777	IMR
4.2.2	**353766**	T->C	228I	*eccD3*	Rv0290	cell wall and cell processes
4.2.2.1	1455780	T->C	96L	*prfA*	Rv1299	information pathways
4.2.2.2	**611463**	G->A	204T	*Rv0519c**	Rv0519c	cell wall and cell processes
4.3	**1452071**	C->A	25G	*thrB*	Rv1296	IMR
4.3.1	**825585**	T->C	262Y	*secY*	Rv0732	cell wall and cell processes
4.3.1.1	**1647807**	T->C	273I	*Rv1461*	Rv1461	conserved hypotheticals
4.3.2	**3414791**	G->C	56A	*nrdH*	Rv3053c	information pathways
4.3.2.1	**784581**	G->C	699T	*fusA1*	Rv0684	information pathways
4.3.3	**2077253**	G->A	459T	*gcvB*	Rv1832	IMR
4.3.4	**1297327**	G->A	392V	*lpqW*	Rv1166	cell wall and cell processes
4.3.4.1	**1274335**	G->A	327L	*mmpL13b**	Rv1146	cell wall and cell processes
4.3.4.2	**784440**	G->T	652A	*fusA1*	Rv0684	information pathways
4.3.4.2.1	**225495**	T->C	359V	*Rv0193c**	Rv0193c	conserved hypotheticals
4.4	4307886	G->A	343R	*serS*	Rv3834c	information pathways
4.4.1	**2905505**	G->A	196T	*hisS*	Rv2580c	information pathways
4.4.1.1	355181	G->A	228K	*mycP3*	Rv0291	IMR
4.4.1.1.1	**15036**	C->G	41A	*trpG*	Rv0013	IMR
4.4.1.2	**342201**	C->G	24P	*eccA3*	Rv0282	cell wall and cell processes
4.4.2	**985287**	G->A	495P	*fprB**	Rv0886	IMR
4.5	**620029**	C->T	47L	*ccsA*	Rv0529	IMR
4.6	**18091**	G->A	224T	*pknA*	Rv0015c	regulatory proteins
4.6.1	4260268	G->C	293A	*pks13*	Rv3800c	lipid metabolism
4.6.1.1	**4406749**	G->A	261L	*parA*	Rv3918c	cell wall and cell processes
4.6.1.2	**1098698**	C->G	397G	*mprB*	Rv0982	regulatory proteins
4.6.2	**4260742**	G->A	135P	*pks13*	Rv3800c	lipid metabolism
4.6.2.1	**896119**	C->T	100F	*purL*	Rv0803	IMR
4.6.2.2	2875883	C->T	201L	*alaS*	Rv2555c	information pathways
4.6.3	**734562**	G->A	103K	*nusG*	Rv0639	information pathways
4.6.4	**4236903**	G->A	375A	*dprE1*	Rv3790	lipid metabolism
4.6.5	**17665**	G->A	366N	*pknA*	Rv0015c	regulatory proteins
4.7	**716918**	G->A	85T	*vapC30**	Rv0624	VDA
4.8	**1130526**	G->A	112A	*ispE*	Rv1011	IMR
4.8.1	**2914906**	G->C	277T	*secD*	Rv2587c	cell wall and cell processes
4.8.2	**2417281**	G->A	65Y	*murX*	Rv2156c	cell wall and cell processes
4.8.3	**616408**	C->G	62A	*Rv0525*	Rv0525	conserved hypotheticals
4.9	**420008**	A->G	58A	*dnaK*	Rv0350	VDA
4.9.1	**119600**	C->G	629V	*Rv0102*	Rv0102	cell wall and cell processes
5	1799921	C->A	113G	*hisD*	Rv1599	IMR
6	**982363**	G->T	64G	*serC*	Rv0884c	IMR
7	1137518	G->A	181N	*glmU*	Rv1018c	cell wall and cell processes

8	**221190**	G->T	178V	*ilvD*	Rv0189c	IMR
9	**2750052**	G->A	58T	*valS*	Rv2448c	information pathways
M.bovis	**62768**	A->G	791G	*dnaB*	Rv0058	information pathways
M.caprae	**904090**	T->C	122G	*purM*	Rv0809	IMR
M.orygis	**44812**	G->T	417P	*leuS*	Rv0041	information pathways

All essential genes (except *) with synonymous changes; Bolded - different from reference [1]; IMR = Intermediary metabolism and respiration; VDA = virulence, detoxification, adaptation

(a) Principal component (PC) analysis plot for lineages 1-9, *M. bovis, M. caprae, M. orygis* (90 sub-lineages; N=35,298)

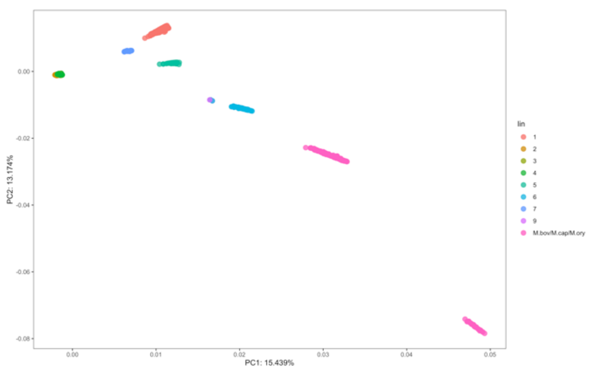

(d) Lineage 3 (7 sub-lineages; N=2,654*)

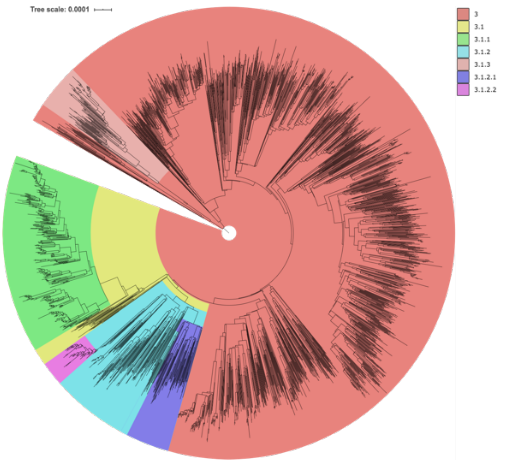

(b) Lineage 1 (16 sub-lineages; N=2,162*)

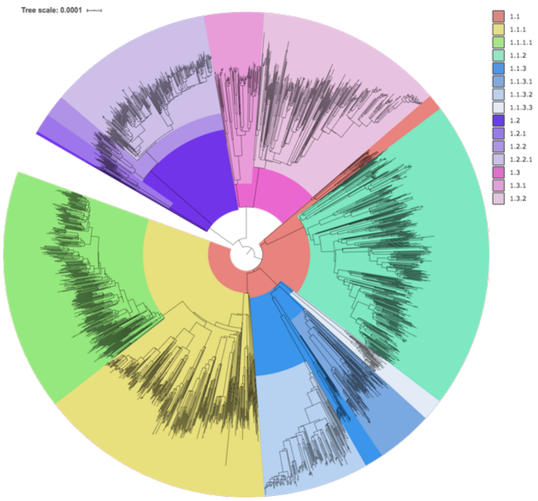

(e) Lineage 4 (52 sub-lineages; N=8,320*)

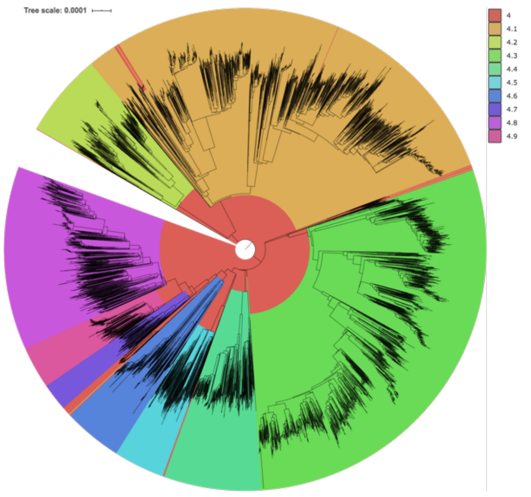

(c) Lineage 2 (7 sub-lineages; N=4,456*)

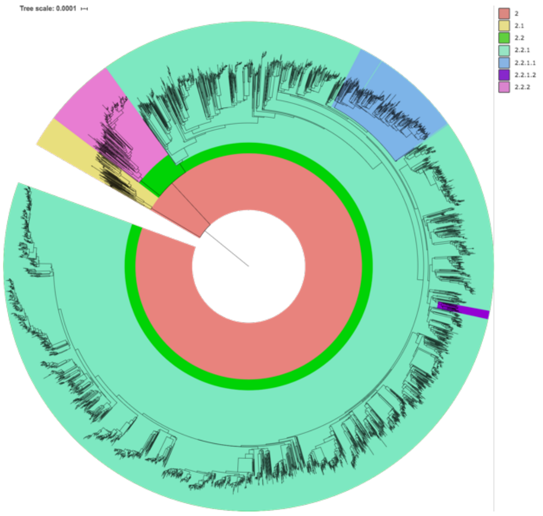

(f) Lineages 5-9, *M.bovis, caprae,* and *orygis**

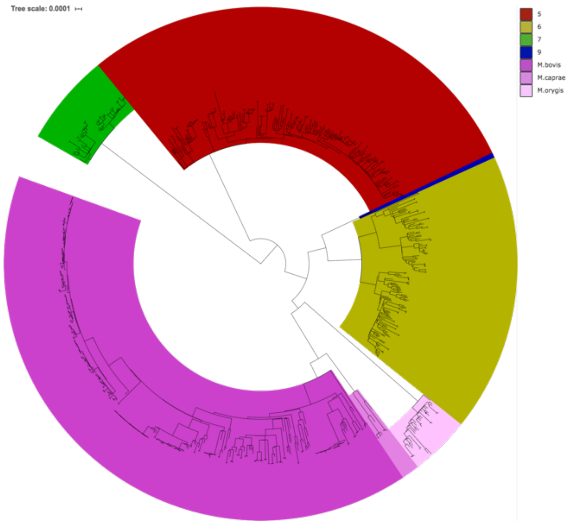

Fig S1: Population structure of the *Mycobacterium tuberculosis* complex isolates by lineage; * training

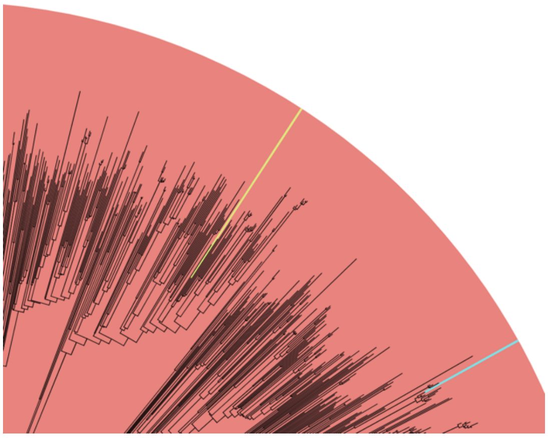

(a) Samples labelled as belonging to 3.1 (yellow) and 3.1.2 (blue) are embedded in the parental clade 3 (red); these samples were therefore re-assigned to the parent clade 3.

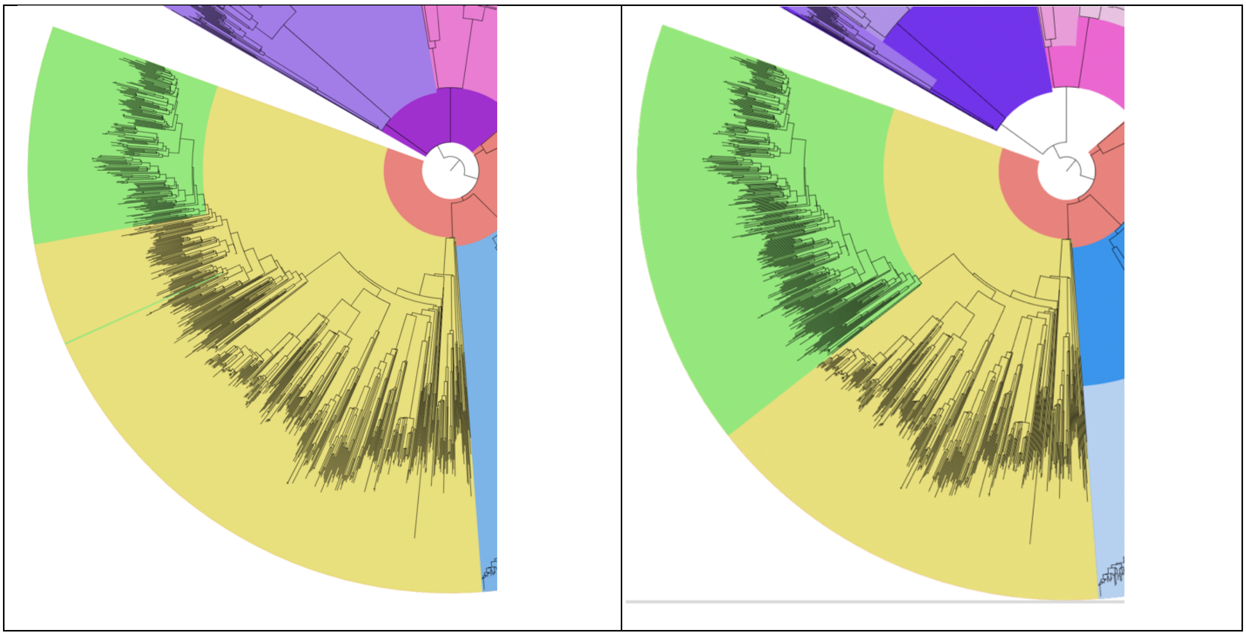

(b) Samples originally labelled 1.1.1.1 (green, left) are within a clade deeper in the tree than they ought to be. The long central branch in the middle of the 1.1.1 (yellow) parent clade suggests a more natural position for this lineage, as shown in (right)

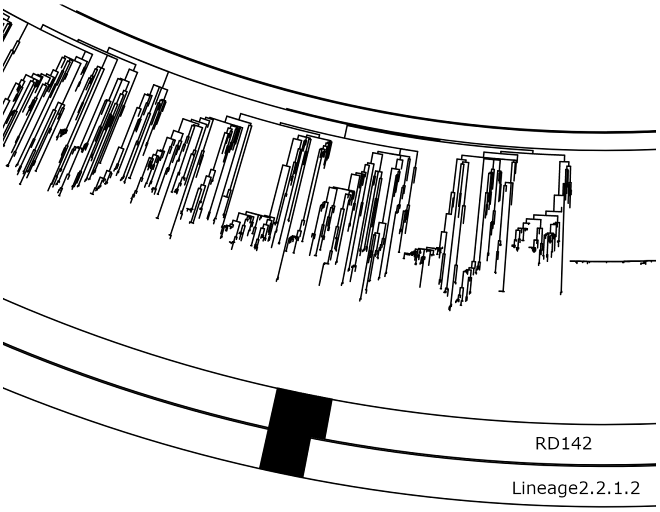

(c) Lineage 2.2.1.2 was defined to reflect the strains containing the RD142 deletion. The original barcode SNP did not capture all strains with this RD and needed to be redefined

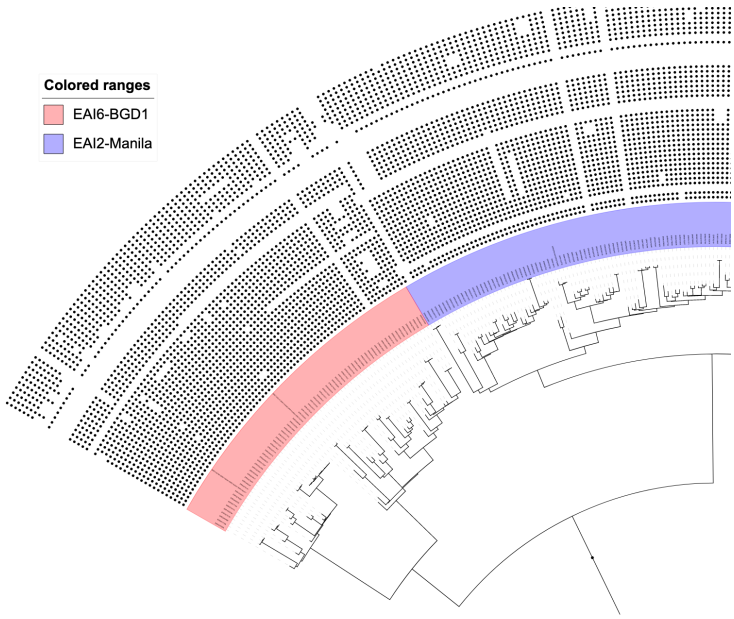

(d) Lineage 1.2.1 encapsulated a large clade with low imbalance and late diversification timing, which was further partitioned into lineage 1.2.1 and 1.2.2, which now reflect members of the EAI6-BGD1 and EAI2-Manila/EAI2-Nonthaburi families, respectively

Fig S2 (a)-(d): Examples of discrepancies using the 62-SNP barcode

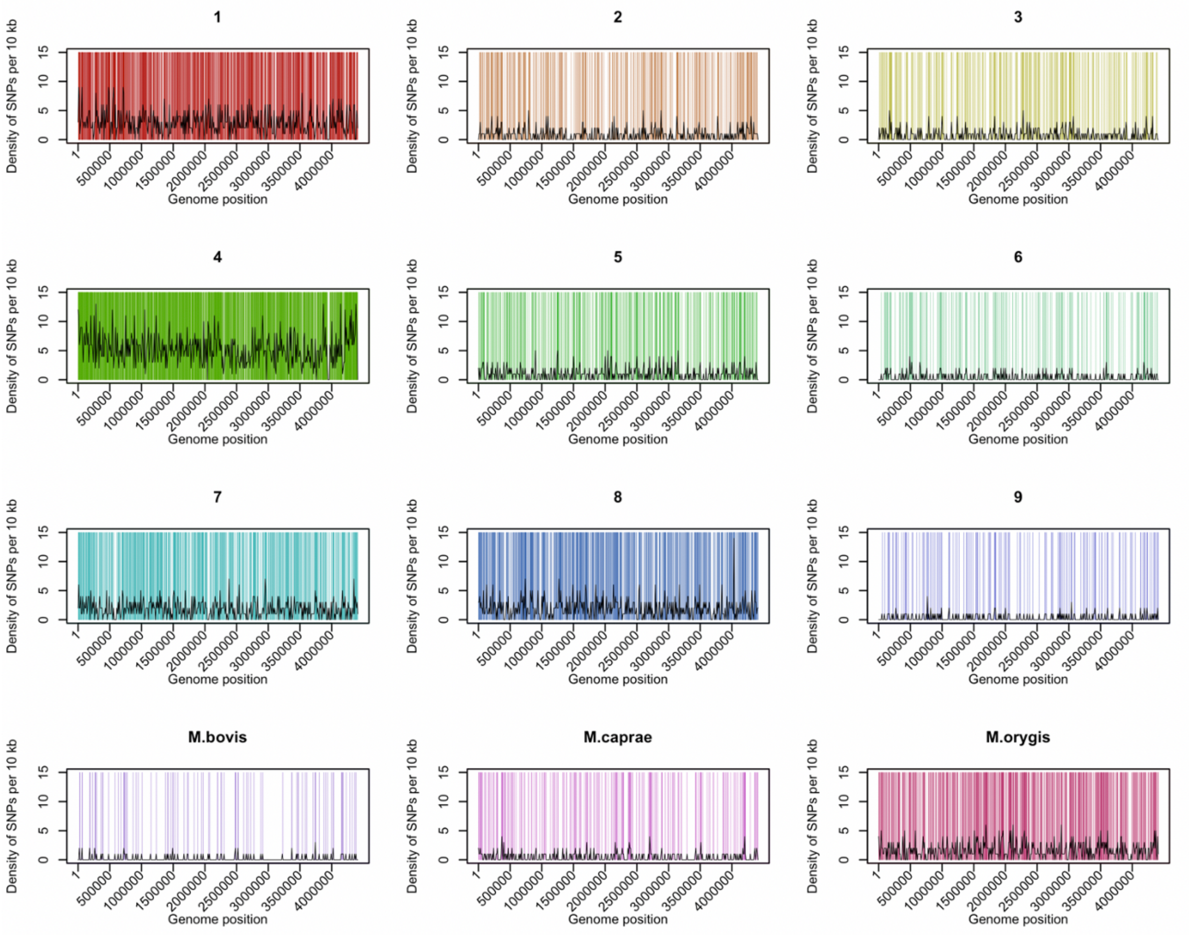

Fig S3: The genome-wide distribution and density (per 10kb) of barcoding SNPs (F_{ST}=1) for each lineage

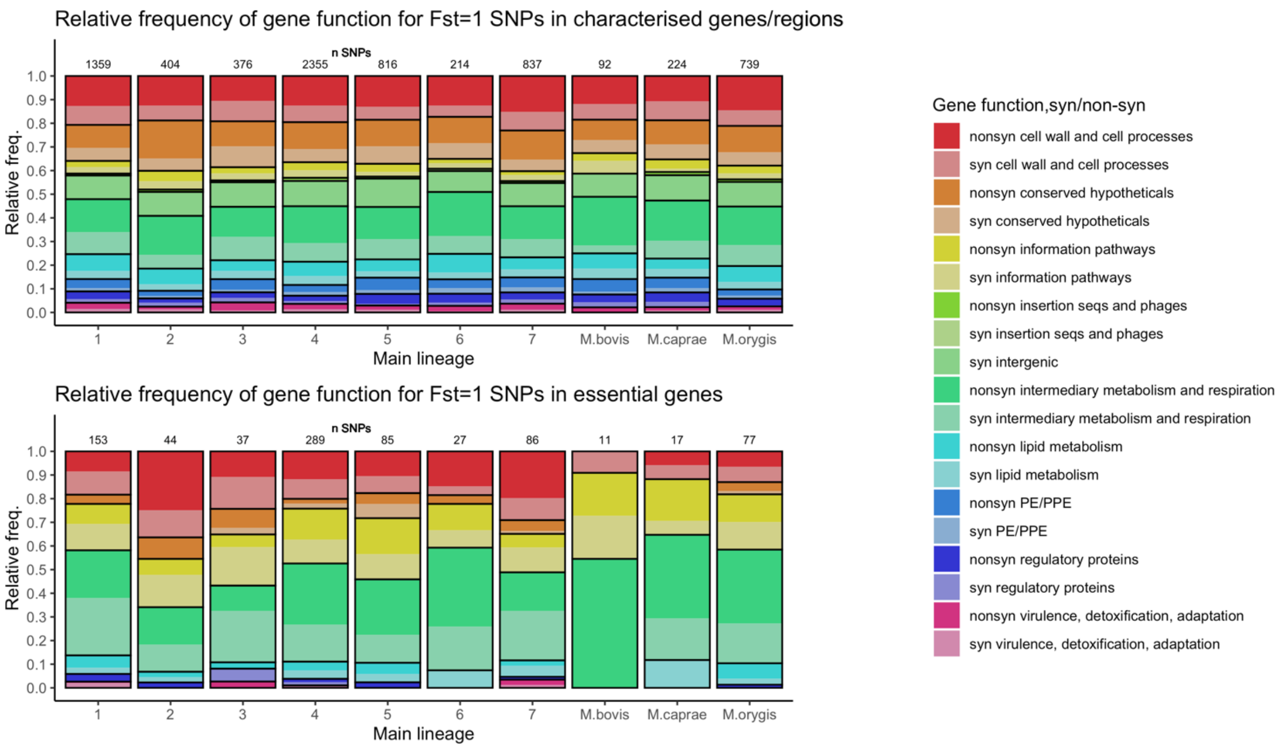

Fig S4: Functional differences between genes containing lineage-barcoding (F_{ST}=1) SNPs*

* Lineages 8 (N=2) and 9 (N=3) have been excluded due to their small sample sizes

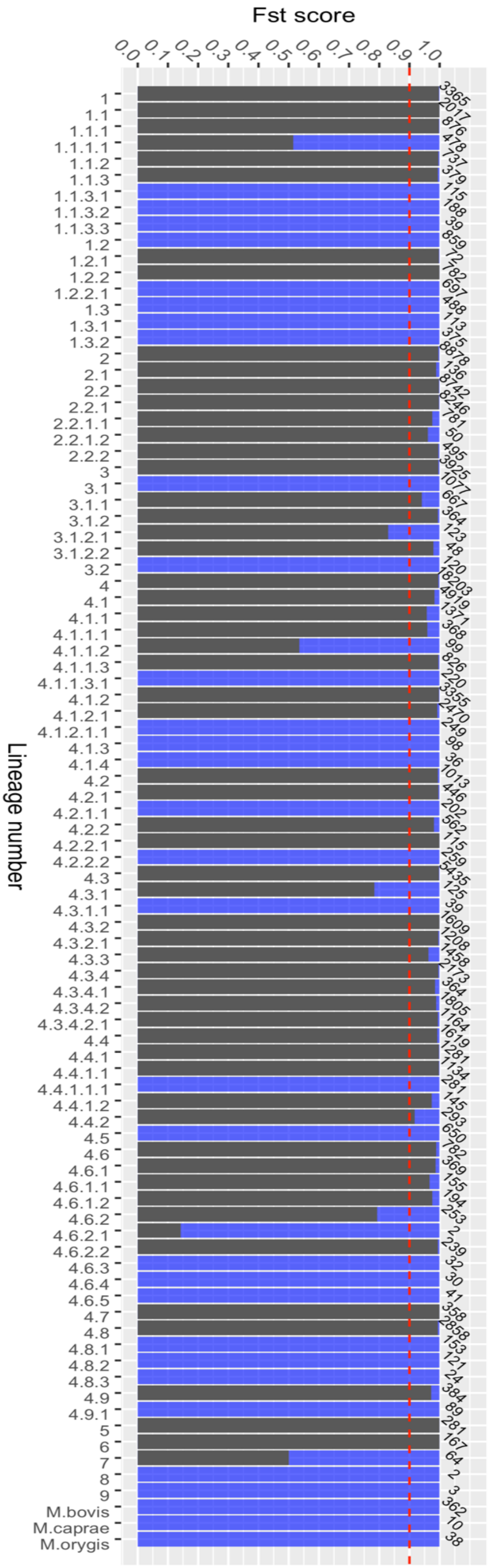

Fig S5: Differentiation of sub-lineages* when comparing the 62- versus 90-SNP barcodes
Blue is the incremental improvement in the new 90-SNP barcode; * based on the F_{ST} measure where values of 1 mean that the barcoding SNPs perfectly differentiate the sub-lineage (versus any other); sub-lineage 4.5 is entirely blue, because we identified a new barcoding SNP which did not lie within a genomic region containing a common deletion

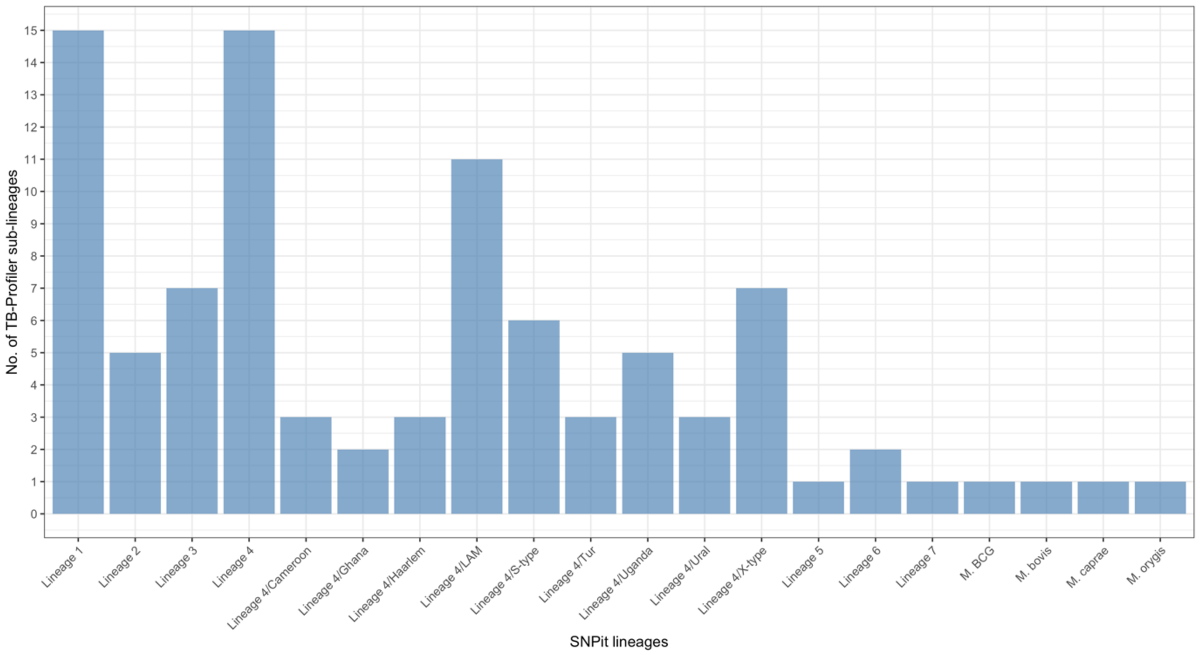

Fig S6: The increased resolution of our 90-SNP barcode (implemented in *TB-Profiler* software) over the comparable (sub-)lineages of the *SNP-IT* tool
